# Suppression of inositol pyrophosphate toxicosis and hyper-repression of the fission yeast *PHO* regulon by loss-of-function mutations in chromatin remodelers Snf22 and Sol1

**DOI:** 10.1128/mbio.01252-24

**Published:** 2024-06-20

**Authors:** Beate Schwer, Aleksei Innokentev, Ana M. Sanchez, Angad Garg, Stewart Shuman

**Affiliations:** 1 Department of Microbiology and Immunology, Weill Cornell Medical College, New York, New York, USA; 2 Molecular Biology Program, Memorial Sloan Kettering Cancer Center, New York, New York, USA; 3 Gerstner Sloan Kettering Graduate School of Biomedical Sciences, New York, New York, USA; Duke University, Durham, North Carolina, USA

**Keywords:** PHO gene expression, transcriptional regulation, fission yeast, Snf22, inositol pyrophosphate toxicosis

## Abstract

Inositol pyrophosphates are signaling molecules that regulate cellular phosphate homeostasis in eukaryal taxa. In fission yeast, where the phosphate regulon (comprising phosphate acquisition genes *pho1*, *pho84*, and *tgp1*) is repressed under phosphate-replete conditions by lncRNA-mediated transcriptional interference, mutations of inositol pyrophosphatases that increase IP_8_ levels derepress the *PHO* regulon by eliciting precocious termination of lncRNA transcription. Asp1 pyrophosphatase mutations resulting in too much IP_8_ are cytotoxic in YES medium owing to overexpression of glycerophosphodiester transporter Tgp1. IP_8_ toxicosis is ameliorated by mutations in cleavage/polyadenylation and termination factors, perturbations of the Pol2 CTD code, and mutations in SPX domain proteins that act as inositol pyrophosphate sensors. Here, we show that IP_8_ toxicity is alleviated by deletion of *snf22*^+^, the gene encoding the ATPase subunit of the SWI/SNF chromatin remodeling complex, by an ATPase-inactivating *snf22-*(*D996A-E997A*) allele, and by deletion of the gene encoding SWI/SNF subunit Sol1. Deletion of *snf22^+^* hyper-repressed *pho1* expression in phosphate-replete cells; suppressed the *pho1* derepression elicited by mutations in Pol2 CTD, termination factor Seb1, Asp1 pyrophosphatase, and 14-3-3 protein Rad24 (that favor precocious *prt* lncRNA termination); and delayed *pho1* induction during phosphate starvation. RNA analysis and lack of mutational synergies suggest that Snf22 is not impacting 3′-processing/termination. Using reporter assays, we find that Snf22 is important for the activity of the *tgp1* and *pho1* promoters, but not for the promoters that drive the synthesis of the *PHO*-repressive lncRNAs. Transcription profiling of *snf22*∆ and *snf22-*(*D996A-E997A*) cells identified an additional set of 66 protein-coding genes that were downregulated in both mutants.

**IMPORTANCE:**

Repression of the fission yeast *PHO* genes *tgp1*, *pho1*, and *pho84* by lncRNA-mediated interference is sensitive to inositol pyrophosphate dynamics. Cytotoxic *asp1-STF* alleles derepress the *PHO* genes via the action of IP_8_ as an agonist of precocious lncRNA 3′-processing/termination. IP_8_ toxicosis is alleviated by mutations of the Pol2 CTD and the 3′-processing/termination machinery that dampen the impact of toxic IP_8_ levels on termination. In this study, a forward genetic screen revealed that IP_8_ toxicity is suppressed by mutations of the Snf22 and Sol1 subunits of the SWI/SNF chromatin remodeling complex. Genetic and biochemical evidence indicates that the SWI/SNF is not affecting 3′-processing/termination or lncRNA promoter activity. Rather, SWI/SNF is critical for firing the *PHO* mRNA promoters. Our results implicate the ATP-dependent nucleosome remodeling activity of SWI/SNF as necessary to ensure full access of *PHO*-activating transcription factor Pho7 to its binding sites in the *PHO* mRNA promoters.

## INTRODUCTION

Inositol pyrophosphates (IPPs) are key effectors of eukaryal phosphate homeostasis, a transcriptional program in which the expression of genes involved in phosphate mobilization and transport is responsive to phosphate availability (1–6). IPPs affect transcription in distinct ways in different taxa. In the fission yeast *Schizosaccharomyces pombe*, a phosphate homeostasis (*PHO*) regulon (7) comprising three phosphate acquisition genes—*pho1* (cell surface acid phosphatase), *pho84* (inorganic phosphate transporter), and *tgp1* (glycerophosphodiester transporter)—is repressed under phosphate-replete conditions by 5′ flanking lncRNAs *prt* (*ncpho1*), *prt2*, and *nctgp1*, respectively (8). lncRNA transcription across the *PHO* mRNA promoters is thought to displace or prevent access of the activating transcription factor Pho7 to its DNA binding site(s) upstream of the TATA box (9–11) and thereby interfere with *PHO* gene expression. *PHO* mRNA transcription is turned on during phosphate starvation when lncRNA synthesis ebbs (12). The *PHO* regulon is derepressed in phosphate-replete cells by a variety of genetic maneuvers that favor precocious lncRNA 3′-processing/termination in response to poly(A) signals upstream of the mRNA promoters (13, 14). *PHO* lncRNA termination is subject to metabolite control by inositol pyrophosphate 1,5-IP_8_, which acts as an agonist of precocious 3′-processing/termination (15).

IP_8_ dynamics are dictated by kinase and pyrophosphatase enzymes, Asp1 and Aps1, that install and remove the β-phosphate groups (2, 16). Asp1 is a bifunctional enzyme composed of an N-terminal kinase domain that converts 5-IP_7_ to 1,5-IP_8_ and a C-terminal pyrophosphatase domain that converts 1,5-IP_8_ back to 5-IP_7_ (17–20). Asp1 can also phosphorylate IP_6_ to yield 1-IP_7_ and dephosphorylate 1-IP_7_ back to IP_6_. An *asp1*∆ null allele or a kinase-dead *asp1-D333A* allele eliminates intracellular IP_8_ and 1-IP_7_ and increases the level of 5-IP_7_, whereas a pyrophosphatase-defective *asp1-H397A* allele increases the level of IP_8_ (17, 18). Aps1 is a Nudix-family IPP pyrophosphatase (21).

Increasing IP_8_ in *asp1-H397A* cells derepresses the *PHO* regulon and prompts precocious termination of *prt* lncRNA synthesis, in a manner dependent on the multi-subunit cleavage and polyadenylation factor (CPF) complex and transcription termination factor Rhn1. Simultaneous inactivation of the Asp1 and Aps1 IPP pyrophosphatases is synthetically lethal, signifying that too much IP_8_ is toxic, but this lethality is suppressed by mutations of CPF subunits (15). Failure to synthesize IP_8_ via Asp1 kinase active site mutation D333A results in *pho1* hyper-repression, as do CPF subunit mutations. Findings of synthetic lethality of *asp1*∆ (no IP_8_) with CPF subunit mutations argue that IP_8_ plays an important role in essential 3′-processing/termination events, albeit in a manner genetically redundant to CPF (15).

lncRNA control of *PHO* gene expression is also governed by the RNA polymerase II (Pol2) CTD code, a set of instructions inscribed within the C-terminal tandem Y^1^S^2^P^3^T^4^S^5^P^6^S^7^ repeat array of the Rpb1 subunit via dynamic phosphorylation/dephosphorylation of the hydroxyamino acids of the CTD and *cis–trans* isomerization of the prolines. Alanine substitution of all CTD Thr4 positions prevents installation of Thr-PO_4_ mark and results in *pho1* hyper-repression. By contrast, mutating all Ser7 residues to alanine elicits *pho1* derepression, as do chimeric CTD mutations in which Ser5 or Pro6 is changed to alanine in every other heptad of the repeat array (13, 22). A forward genetic screen for mutations that derepress Pho1 acid phosphatase expression in *CTD-T4A* cells (23) yielded 18 independent *STF* (suppressor of threonine four) isolates, every one of which had a mutation in the C-terminal pyrophosphatase domain of Asp1. The *STF6* and *STF9* mutations—*asp1-386*(*Stop*) and *asp1-493*(*Stop*), respectively—were lethal in a wild-type CTD background but were viable when combined with deletion or loss-of-function mutations in CPF subunits and Rhn1 (23). These findings implicated Asp1 pyrophosphatase in constraining IP_8_ synthesis by Asp1 kinase, without which IP_8_ can accumulate to toxic levels that elicit precocious termination by CPF/Rhn1.

Extending the screen for suppression of transcriptional interference with *pho1* identified a mutation, G476S, in the RNA-binding domain of the essential termination factor Seb1 that evokes precocious *prt* and *nctgp1* lncRNA termination and derepresses *pho1* in a manner dependent on CPF/Rhn1 and IP_8_ synthesis (24). The *seb1-G476S* allele was synthetically lethal in the absence of inositol pyrophosphatase Aps1; the synthetic lethality was rescued by CPF/Rhn1 loss-of-function alleles (24).

Because the mechanism of IP_8_ toxicosis and the signaling pathway(s) that connect IP_8_ elevation to Pol2 termination were uncertain, we initiated a genetic screen for spontaneous mutations that suppressed the toxicity of inositol pyrophosphatase mutations. For this purpose, we exploited three *STF* alleles of *asp1—STF3* (*G863D*), *STF5* (*C643Y*), and *STF7* (*H686Y*)— that caused severe growth defects *per se* on standard yeast extract with supplements (YES) medium. Via this *SST* (suppressor of suppressor of Thr4) screen, we identified a missense mutation of the essential Cft1 subunit of CPF that sufficed to suppress even the lethal Asp1 pyrophosphatase mutations *STF6* and *STF9* (25). This result reinforced the case for CPF as a target of IP_8_ toxicosis. The initial *SST* screen also identified two new agents of fission yeast IPP transactions, Gde1 and Spx1 (also known as Pqr1), both of which have an IPP-binding SPX domain (3, 26) and both of which are required for IP_8_ toxicity (25).

We had assumed until recently that IP_8_ toxicity on standard YES medium ensues from dysregulation of 3′-processing/termination of one or more essential fission yeast genes. A new and instructive observation was that the growth defect of *asp1-STF* mutants is caused by a titratable constituent in yeast extract and that all *asp1-STF* mutants grew well on the synthetic medium, ePMGT (enhanced Pombe Minimal Glutamate with Thiamine) (27). Via a genetic screen for new spontaneous suppressors, we identified a null mutation of glycerophosphodiester transporter Tgp1 that abolished *asp1-STF* toxicity on YES medium. This result, and the fact that *tgp1* mRNA expression is increased by >40-fold in *asp1-STF* cells, led to the discovery that (i) glycerophosphocholine (GPC) recapitulates the toxicity of yeast extract to *asp1-STF* cells in a Tgp1-dependent manner and (ii) induced overexpression of *tgp1* in *asp1*^+^ wild-type cells or *asp1*∆ cells (lacking IP_8_) also elicits toxicity dependent on GPC (27). Thus, IP_8_ toxicity in *asp1-STF* cells is a consequence of derepression of the nonessential *tgp1* gene, which causes increased uptake of GPC. It is conceivable that elevated levels of intracellular GPC are toxic *per se* (e.g., by perturbing phospholipid dynamics) or that elevated GPC engenders its conversion into elevated levels of derivatives that are toxic.

In the present study, we extend the *SST* screen and thereby report that mutations of the Snf22 ATPase subunit and the Sol1 subunit of the SWI/SNF chromatin remodeling complex rescue the toxicity of *asp1-STF* mutants. The absence of Snf22, or an ATPase-inactivating alanine mutation of Snf22, results in hyper-repression of the *PHO* genes in phosphate-replete cells and interdicts the *pho1* derepressive effects of inositol pyrophosphatase, Pol2 CTD, Seb1, and Rad24 mutations that act via enhancement of precocious *prt* lncRNA 3′-processing/termination. Yet, we find that the effect of *snf22* inactivation is not exerted at the level of 3′-processing/termination. Rather, the Snf22 function (and by inference chromatin remodeling) is important for the activity of the *tgp1* and *pho1* mRNA promoters. We attribute the suppression of *asp1-STF* toxicity by *snf22* mutations to a dampening of the *tgp1* derepression that underlies IP_8_ toxicity.

## RESULTS

### Isolation of a *snf22* mutant in a screen for spontaneous suppressors of IP_8_ toxicosis

**Fig 1:**
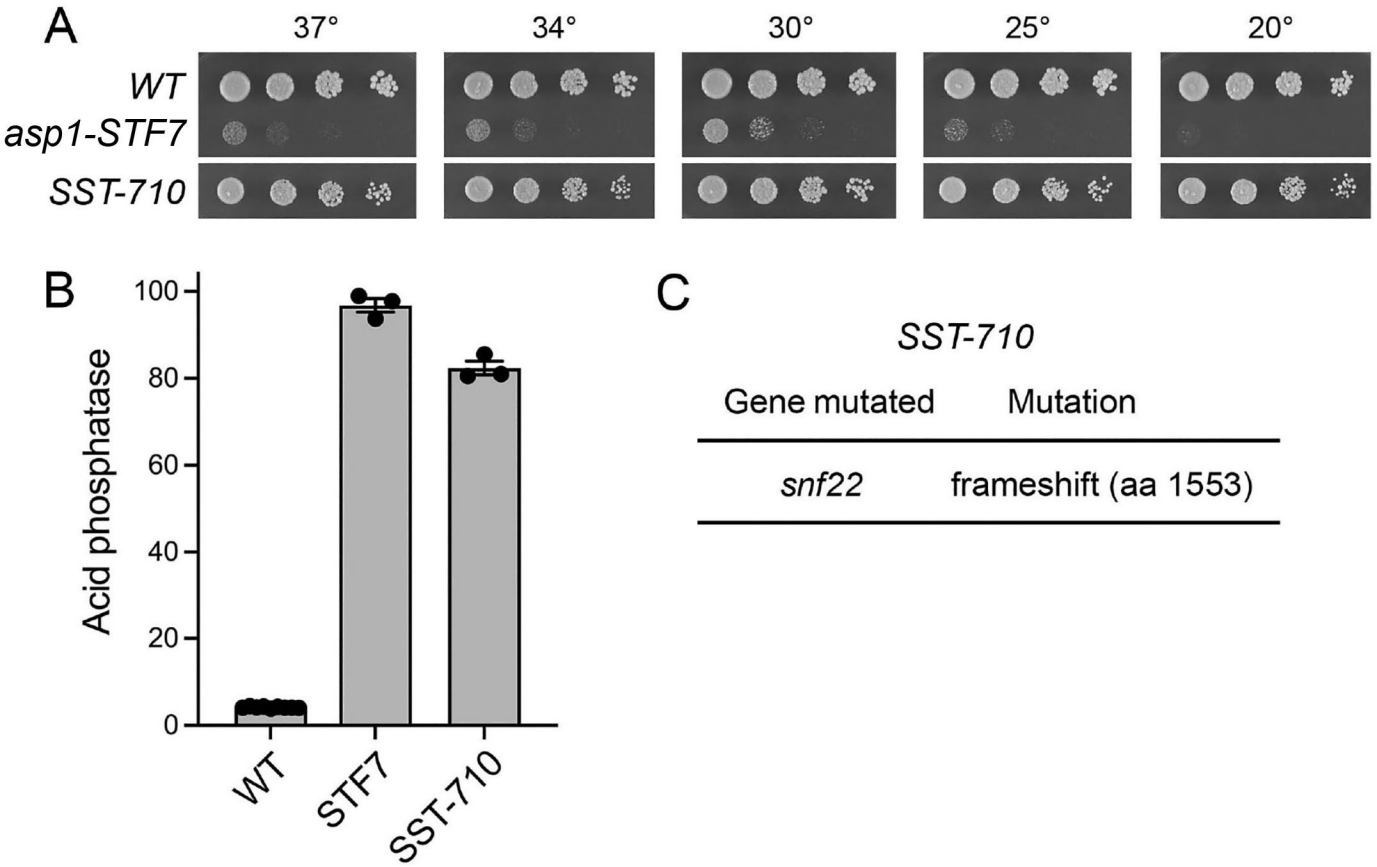
Isolation of a *snf22* mutant in a screen for spontaneous suppressors of IPP toxicosis. (**A**) Serial fivefold dilutions of wild-type, *asp1-STF7*, and *SST-710* cells were spot-tested for growth on the same YES agar plate at the indicated temperatures. The white line between the *asp1-STF7* and *SST-710* spottings indicates that irrelevant segments of the photographs of the plates were cropped in preparing the figure. (**B**) The indicated strains were grown to *A*_600_ of 0.5 to 0.8 in liquid culture in YES medium at 30°C. Cells were then harvested, washed with water, and assayed for Pho1 acid phosphatase activity by conversion of *p*-nitrophenylphosphate to *p*-nitrophenol. Activity is expressed as the ratio of *A*_410_ (*p*-nitrophenol production) to *A*_600_ (input cells). (**C**) Whole-genome sequencing of the *SST-710* strain revealed the indicated *snf22* mutation.

The *STF7* (*H686Y*) pyrophosphatase domain mutation of *asp1* elicits a severe growth defect at all temperatures (Fig. 1A). We screened for candidate *SST* (suppressors of STF) mutants by plating *STF7* cells on YES agar at 30°C and selecting rare single colonies that grew to large size against a background of tiny colonies. These were grown and re-streaked for single colonies, which were homogeneously larger than the parental *STF7* mutant. In our initial report describing the *SST* screen, we isolated strain *SST-75* as a spontaneous suppressor of *STF7* that grew as well as wild type on YES agar at 25 to 37°C and we showed that suppression was caused by a Cys823Arg missense mutation in the essential Cft1 subunit of CPF (25). Here we focus on the *SST-710* strain (a suppressor of *STF7* isolated in the same screen that yielded *SST-75*) that grew well on YES agar at all temperatures, as gauged by colony number, albeit slightly slower than wild type based on colony size (Fig. 1A). To exclude the possibility that the *STF* suppression resulted from reversion of the original *asp1-STF* missense mutation, or from a kinase-inactivating mutation in the N-terminal kinase domain of Asp1, we amplified and sequenced the *asp1* ORF from *SST-710* and verified that the original *STF7* allele of *asp1* was unchanged.

We assayed the wild-type, *STF7*, and *SST-710* strains for cell-surface associated Pho1 acid phosphatase activity (Fig. 1B). The *asp1-STF7* pyrophosphatase mutation resulted in derepression of Pho1 expression, by 23-fold compared to the wild-type control. The derepression was slightly attenuated in the *STF-710* strain, by 15% compared to the parental *STF7* strain (Fig. 1B); this difference was statistically significant (*P* value of 0.003 by unpaired *t*-test).

Paired-end Illumina sequencing of unamplified genomic DNA from the *SST-710* strain was performed to achieve at least 100-fold coverage of the fission yeast genome. The *SST-710* genome was compared to that of the parental *STF7* mutant that we sequenced previously (23). The *SST-710* strain contains a single frameshift mutation in the *snf22* gene (Fig. 1C), which is nonessential for fission yeast vegetative growth, and which encodes the 1680-aa ATPase subunit of the fission yeast SWI/SNF chromatin remodeling factor (28). The *SST-710* allele is a +1 frameshift at the codon for Tyr1553 that replaces the native polypeptide following Leu1552 with a Leu-Pro dipeptide, after which the mutant protein terminates at a new in-frame stop codon. According to an AlphaFold model (alphafold.ebi.ac.uk/entry/O94421), the deleted C-terminal segment of Snf22 embraces four α-helices (aa 1554–1649), three of which are constituents of a Bromo domain 4-helix bundle, followed by a disordered region (aa 1650–1680). The Bromo domain is implicated in the recognition of acetylated lysine residues in chromatin (29).

### *snf22*∆ suppresses Pho1 derepression in *asp1-H397A*, *aps1*∆, and *duf89*∆ mutants

**Fig 2:**
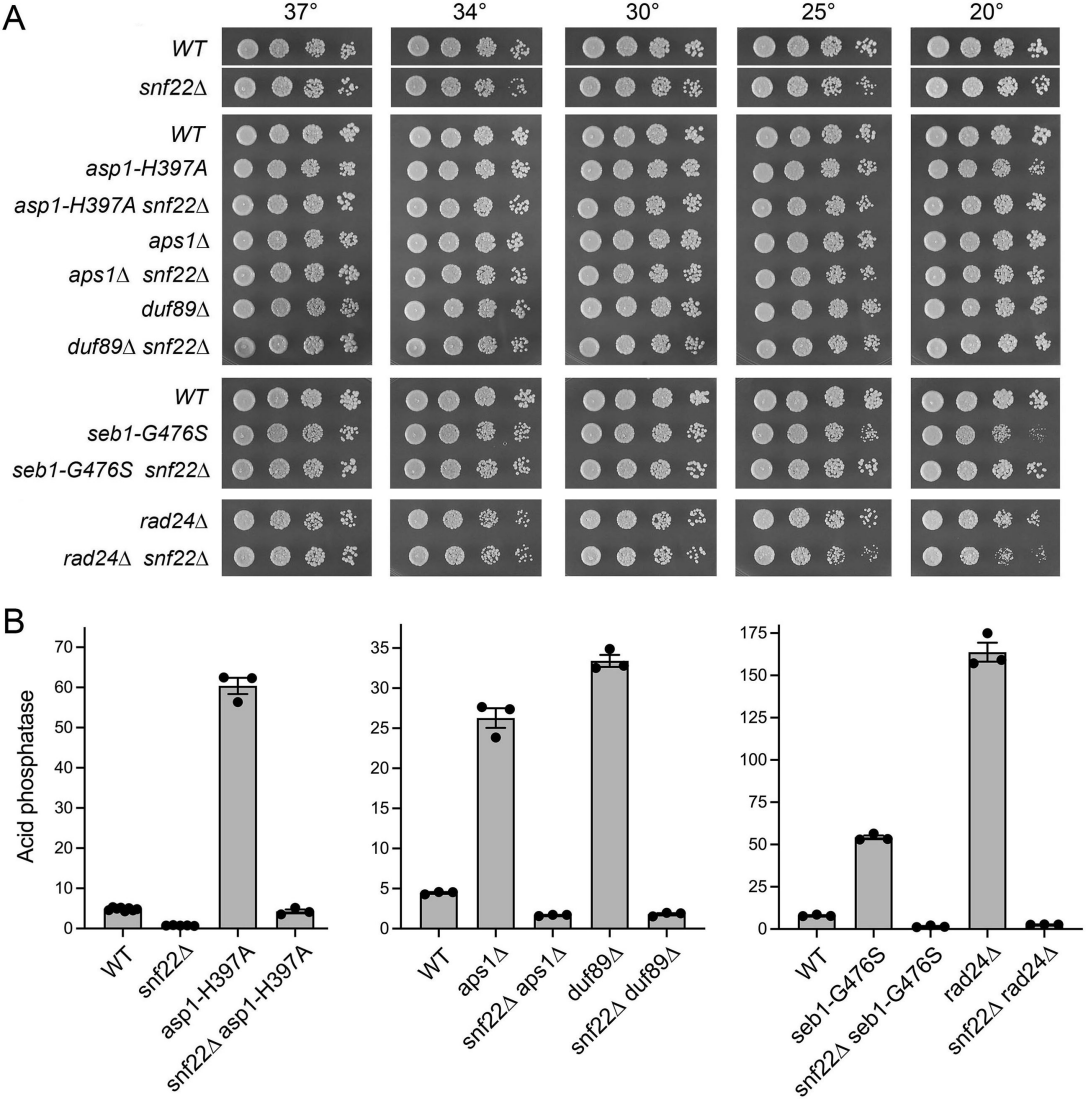
*snf22*∆ suppresses Pho1 derepression by *asp1-H397A*, *aps1*∆, *duf89*∆, *seb1-G476S*, and *rad24*∆. (**A**) Serial fivefold dilutions of fission yeast strains (as specified on the left) were spot-tested for growth on YES agar at the indicated temperatures. (**B**) The indicated strains were assayed for Pho1 acid phosphatase activity.

We constructed a *snf22*∆ strain in which the chromosomal *snf22*^+^ open reading frame encoding Snf22 amino acids 1 to 1213 was deleted and replaced by a *kanMX* drug resistance marker. *snf22*∆ cells grew similarly to wild-type cells on YES agar at all temperatures tested (Fig. 2A). *snf22*∆ elicited a sevenfold hyper-repression of Pho1 expression in phosphate-replete cells compared to the wild-type control (Fig. 2B, *P* value <0.0001). We proceeded to test the effect of *snf22*∆ in the *asp1-H397A* strain background, wherein IP_8_ levels are elevated (17), though not to the point of toxicity, and Pho1 expression is derepressed (Fig. 2B). The *snf22*∆ *asp1-H397A* double mutant grew well on YES agar at all temperatures from 20°C to 37°C (Fig. 2A). The derepression of Pho1 seen in *asp1-H397A* cells was erased by *snf22*∆, to the extent that acid phosphatase activity in *snf22*∆ *asp1-H397A* cells was the same as that of the wild-type control (Fig. 2B).

Deletion of the Nudix-family pyrophosphatase Aps1 or the Duf89 phosphatase-pyrophosphatase also results in the derepression of Pho1 expression (15, 30), an effect that was suppressed by *snf22*∆ (Fig. 2B). The *snf22*∆ *aps1*∆ and *snf22*∆ *duf89*∆ strains grew well on YES agar at all temperatures tested (Fig. 2A).

### *snf22*∆ suppresses Pho1 derepression elicited by Seb1 and Rad24 mutations

A mutation G476S in the RNA-binding domain of termination factor Seb1 leads to precocious *nctgp1* and *prt* lncRNA termination resulting in derepression of the downstream *tgp1* and *pho1* mRNA promoters (24). Pho1 expression is therefore derepressed in *seb1-G476S* cells (Fig. 2B). Here we introduced the *snf22*∆ allele into the *seb1-G476S* strain and made two instructive findings: (i) *snf22*∆ suppressed the cold-sensitive slow growth phenotype associated with *seb1-G476S* (Fig. 2A) and (ii) *snf22*∆ suppressed the derepression of Pho1 by *seb1-G476S* (Fig. 2B).

The 14-3-3 protein Rad24 is a newly recognized governor of fission yeast phosphate homeostasis *via* its effect on transcription interference (31). Production of full-length interfering *prt* lncRNA is inhibited in *rad24*∆ cells (31) concomitant with increased production of *pho1* mRNA and greatly increased Pho1 activity (as in Fig. 2B). Pho1 derepression by *rad24*∆ depends on CPF, Rhn1, and CTD-Thr4. Combining *rad24*∆ with *asp1-H397A* caused a severe synthetic growth defect that was suppressed by CPF, Rhn1, and CTD-T4A mutations (31). These results established Rad24 as an antagonist of precocious RNA 3′-processing/termination. Here we constructed a *snf22*∆ *rad24*∆ double mutant (Fig. 2A) and found that loss of Snf22 abolished Pho1 derepression by *rad24*∆ (Fig. 2B).

### *snf22*∆ suppresses Pho1 derepression elicited by Pol2 CTD mutations

Pan-substitution of Ser7 with alanine in all CTD heptad derepresses Pho1, as do chimeric CTD mutations in which Ser5 or Pro6 is replaced by alanine in every other heptad (22, 32). The derepression caused by CTD mutations is suppressed by mutations of 3′-processing/termination factors (13, 32). Here we crossed *snf22*∆ to CTD mutants *S7A*, *S5·S5A*, and *P6·P6A* and recovered the respective double-mutant strains after sporulation and selection for drug resistance markers linked to the *snf22*∆ and *rpb1-CTD-Ala* loci. Growth of the single and double mutants on YES agar is shown in Fig. S1A. The *snf22*∆ *S5·S5A* and *snf22*∆ *P6·P6A* strains grew more slowly at 20°C than the component single mutants. *snf22*∆ *S7A* cells formed smaller colonies than *S7A* cells at 25°C to 37°C. The salient findings were that *snf22*∆ abolished the derepression of Pho1 by the *CTD S7A*, *S5·S5A*, and *P6·P6A* alleles (Fig. S1B).

### ATPase active site mutant *snf22-*(*D996A-E997A*)

**Fig 3:**
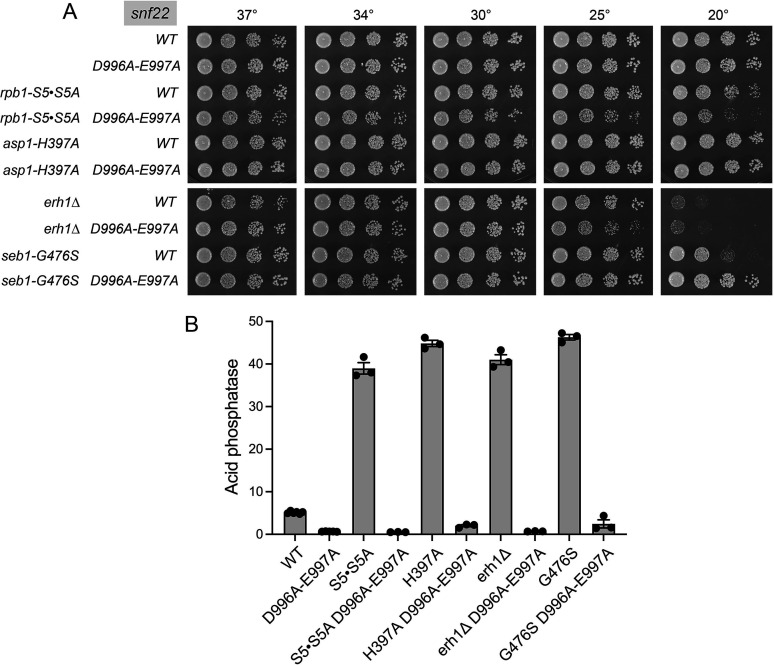
ATPase active site mutant *snf22-*(*D996A-E997A*) blocks Pho1 derepression. (**A**) Serial fivefold dilutions of fission yeast strains (as specified on the left) were spot-tested for growth on YES agar at the indicated temperatures. (**B**) The indicated strains were assayed for Pho1 acid phosphatase activity.

Snf22 belongs to the DExH clade of SF2 NTP phosphohydrolases, so named for the motif II element of the active site (33). The Asp and Glu side chains of the DExH motif serve as ligands for the divalent cation cofactor for NTP hydrolysis (34) and their mutation results in loss of NTPase activity (35, 36). In the case of budding yeast Snf2, a double-alanine mutation of motif II (D894A-E895A) abolishes Snf2 function *in vivo*, as gauged by multiple assays of Snf2-dependent gene expression, without grossly affecting Snf2 protein expression or its assembly into the SWI/SNF complex (37). Here we replaced the wild-type fission yeast chromosomal *snf22*^+^ allele with a mutated version in which Asp996 and Glu997 of the Snf22 ^996^DEGH^999^ motif were both replaced by alanine. The *snf22-*(*D996A-E997A*) strain, which included a drug selection marker 3′ of the *snf22* locus, grew as well as equivalently marked *snf22* wild-type cells on YES agar (Fig. 3A). *snf22-(D996A-E997A*) elicited an eightfold hyper-repression of Pho1 expression in phosphate-replete cells compared to the wild-type control (Fig. 3B, *P* value <0.0001). We constructed a series of double mutants by crossing *snf22-*(*D996A-E997A*) to the *CTD-S5·S5A*, *asp1-H397A*, and *seb1-G476S* strains in which *pho1* expression is derepressed via precocious lncRNA termination. Spot tests of growth on YES agar revealed that *snf22-*(*D996A-E997A*) rescued the cold-sensitive phenotype of *seb1-G476S* (Fig. 3A). The salient findings were that *snf22-*(*D996A-E997A*) suppressed the derepression of Pho1 by *CTD-S5·S5A*, *asp1-H397A*, and *seb1-G476S* (Fig. 3B). Moreover, *snf22-*(*D996A-E997A*) reversed the derepression of Pho1 accompanying deletion of *erh1* (Fig. 3B), which results from precocious 3′-processing/termination of *prt* lncRNA synthesis (38). We conclude that the ATPase activity of Snf22 is required for Snf22 function in alleviating transcriptional interference at the *prt–pho1* locus.

### *snf22-(D996A-E997A*) is a stronger suppressor of IP_8_ toxicosis than *snf22*∆

**Fig 4:**
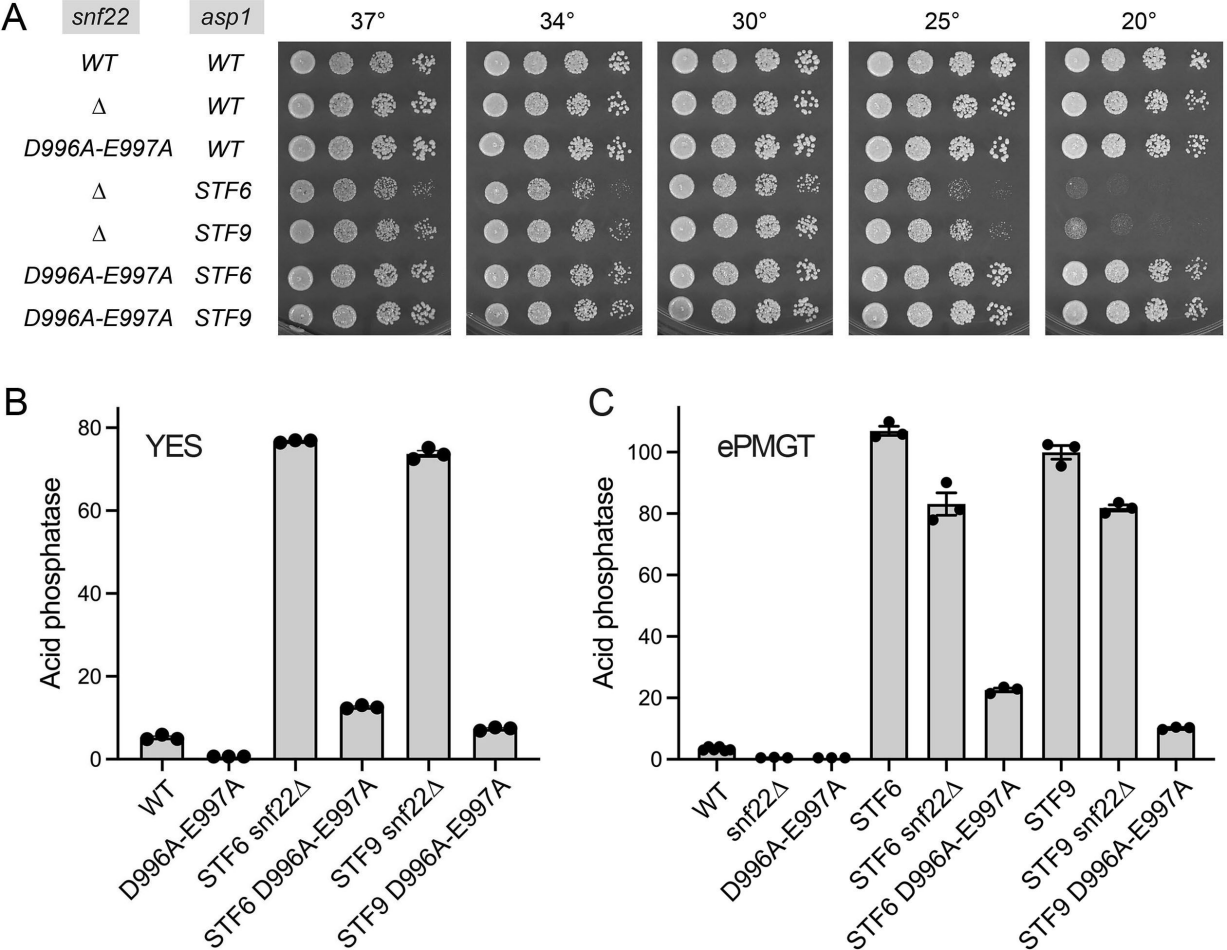
*snf22-*(*D996A-E997A*) is a stronger suppressor of IPP toxicosis than *snf22*∆. (**A**) Serial fivefold dilutions of fission yeast strains (as specified on the left) were spot-tested for growth on YES agar at the indicated temperatures. (**B**) The indicated strains grown in YES medium were assayed for Pho1 acid phosphatase activity. (**C**) The indicated strains grown in ePMGT medium were assayed for Pho1 acid phosphatase activity.

To gauge whether the absence of Snf22 could suppress the lethality of the *STF6* [*asp1-386*(*Stop*)] and *STF9* [*asp1-493*(*Stop*)] mutations that truncate the Asp1 pyrophosphatase domain (23), we mated *snf22*∆ to the *asp1-STF6 CTD-T4A* and *asp1-STF9 CTD-T4A* strains. After sporulation and screening of random spore populations for markers linked to the loci of interest, we obtained viable double-mutant haploid progeny *snf22*∆ *STF6* and *snf22*∆ *STF9*. The *snf22*∆ *STF6* and *snf22*∆ *STF9* mutants grew well at 30°C but were overtly sick at 20°C and slow-growing at 37°C (based on colony size) (Fig. 4A), signifying that not all the deleterious effects of the *STF6* and *STF9* alleles were eliminated in the absence of Snf22. By crossing *snf22-*(*D996A-E997A*) to *STF6 CTD-T4A* and *STF9 CTD-T4A* strains and random spore analysis, we recovered viable *snf22-*(*D996A-E997A*) *STF6* and *snf22-*(*D996A-E997A*) *STF9* haploids that grew well on YES agar at all temperatures tested (Fig. 4A). Thus, the ATPase activity of Snf22 is required for Snf22 function in abetting IP_8_ toxicity and the *snf22-*(*D996A-E997A*) allele was apparently a more potent suppressor of the lethal *STF6* and *STF9* mutations than *snf22*∆. This was affirmed by analyzing Pho1 expression in *snf22-*(*D996A-E997A) STF6* and *snf22-*(*D996A-E997A*) *STF9* cells grown in YES medium, which showed that the ATPase-defective Snf22 mutations reduced acid phosphatase activity back to near wild-type levels, in contrast to the high levels of Pho1 activity seen in *snf22*∆ *STF6* and *snf22*∆ *STF9* cells (Fig. 4B). These results suggest that the ATPase-defective motif II mutant of Snf22 exerts a stronger *in vivo* phenotype than does absence of Snf22.

To better interrogate the strength of *STF* suppression by *snf22* alleles, we took advantage of the recent findings (27) that *asp1-STF* mutants grow well on synthetic ePMGT medium, which lacks yeast extract. We find that cell surface Pho1 acid phosphatase activity in *STF6* and *STF9* cells grown to mid-log phase in ePMGT was 32-fold and 30-fold higher, respectively, than that of *asp1*^+^ wild-type cells (Fig. 4C). The extent of Pho1 derepression was reduced by 22% in *STF6 snf22*∆ cells (*P* value 0.0125) and by 18% in *STF9 snf22*∆ cells (*P* value 0.067) grown in ePMGT (Fig. 4C). The ATPase-defective mutant allele was more potent in this respect, reducing Pho1 derepression by 79% in *STF6 snf22-*(*D996A-E997A*) cells and by 90% in *STF9 snf22-*(*D996A-E997A*) cells grown in ePMGT (Fig. 4C). In *asp1*^+^ wild-type cells grown in ePMGT, the *snf22*∆ and *snf22-*(*D996A-E997A*) alleles reduced Pho1 acid phosphatase activity by 6-fold (*P* value < 0.0001) and sevenfold (*P* value < 0.0001), respectively (Fig. 4C).

**Fig 5:**
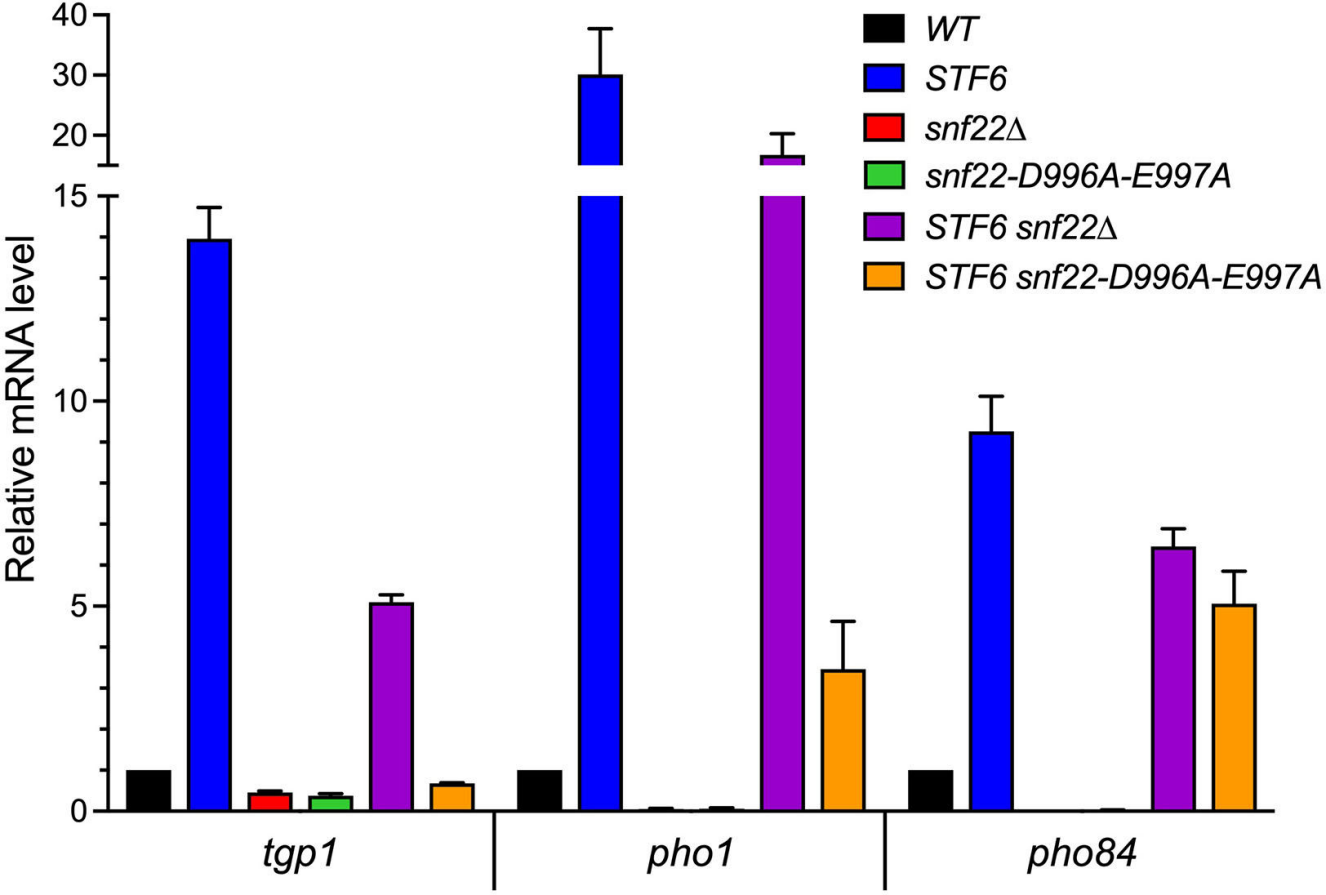
Snf22 is required for derepression of *tgp1* mRNA in *asp1-STF6* cells. Analysis of *tgp1*, *pho1*, and *pho84* mRNA levels in wild-type, *STF6*, *snf22*∆, *snf22-*(*D996A-E997A*), *STF6 snf22∆*, and *STF6 snf22-*(*D996A-E997A*) yeast strains grown to mid-log phase in ePMGT medium was performed by RT-qPCR. The level of each transcript was normalized to that of *act1* mRNA for the same RNA sample. The bar graph shows the relative change in *PHO* mRNA levels in *STF6*, *snf22*∆, *snf22-*(*D996A-E997A*), *STF6 snf22*∆, and *STF6 snf22-*(*D996A-E997A*) relative to the wild-type control (defined as 1.0). Each datum in the graph is the average of RT-qPCR analyses of RNA from three independent yeast cultures; the error bars denote SEM.

Because recent studies revealed that the toxicity of *asp1-STF* mutants in YES medium depends on overexpression of *tgp1* (27), it was of interest to gauge the degree to which the *snf22* suppressor mutations attenuated the derepression of *tgp1* mRNA in *asp1-STF* cells. To do so, we performed *PHO* gene-specific RT-qPCR on total RNA isolated from wild-type, *asp1-STF6*, *snf22*∆, *snf22-*(*D996A-E997A*), *asp1-STF6 snf22*∆, and *asp1-STF6 snf22-*(*D996A-E997A*) cells grown to mid-log phase in ePMGT. The levels of the *tgp1*, *pho1*, and *pho84* mRNAs in the *asp1* and *snf22* mutants were normalized to their respective levels in wild-type cells (defined as 1.0 in Fig. 5). The instructive findings were that the 14-fold increase in *tgp1* mRNA observed in *asp1-STF6* cells was dialed back to a 5-fold increase in *asp1-STF6 snf22*∆ cells and was erased in the *asp1-STF6 snf22-*(*D996A-E997A*) strain (Fig. 5). Similarly, the 30-fold increase in *pho1* mRNA in *asp1-STF6* cells was reduced to a 17-fold increase in *asp1-STF6 snf22*∆ cells and further attenuated to a 3-fold increase in *asp1-STF6 snf22-*(*D996A-E997A*) cells (Fig. 5). Expression of *pho84* mRNA was increased by 9-fold in *asp1-STF6* cells and was less sensitive than *tgp1* or *pho1* to Snf22 loss of function, insofar as *pho84* mRNA remained elevated by sixfold and fivefold in the *asp1-STF6 snf22*∆ and *asp1-STF6 snf22-*(*D996A-E997A*) double mutants, respectively. RT-qPCR analyses also revealed that the *snf22*∆ and *snf22-*(*D996A-E997A*) single mutants elicited a reduction in all three *PHO* mRNAs compared to the wild-type controls: by 2- to 3-fold for *tgp1*; 16- to 19-fold for *pho1*; and 34- to 53-fold for *pho84* (Fig. 5).

### Isolation of a *sol1* mutant as a suppressor of *asp1-STF6* toxicosis

**Fig 6:**
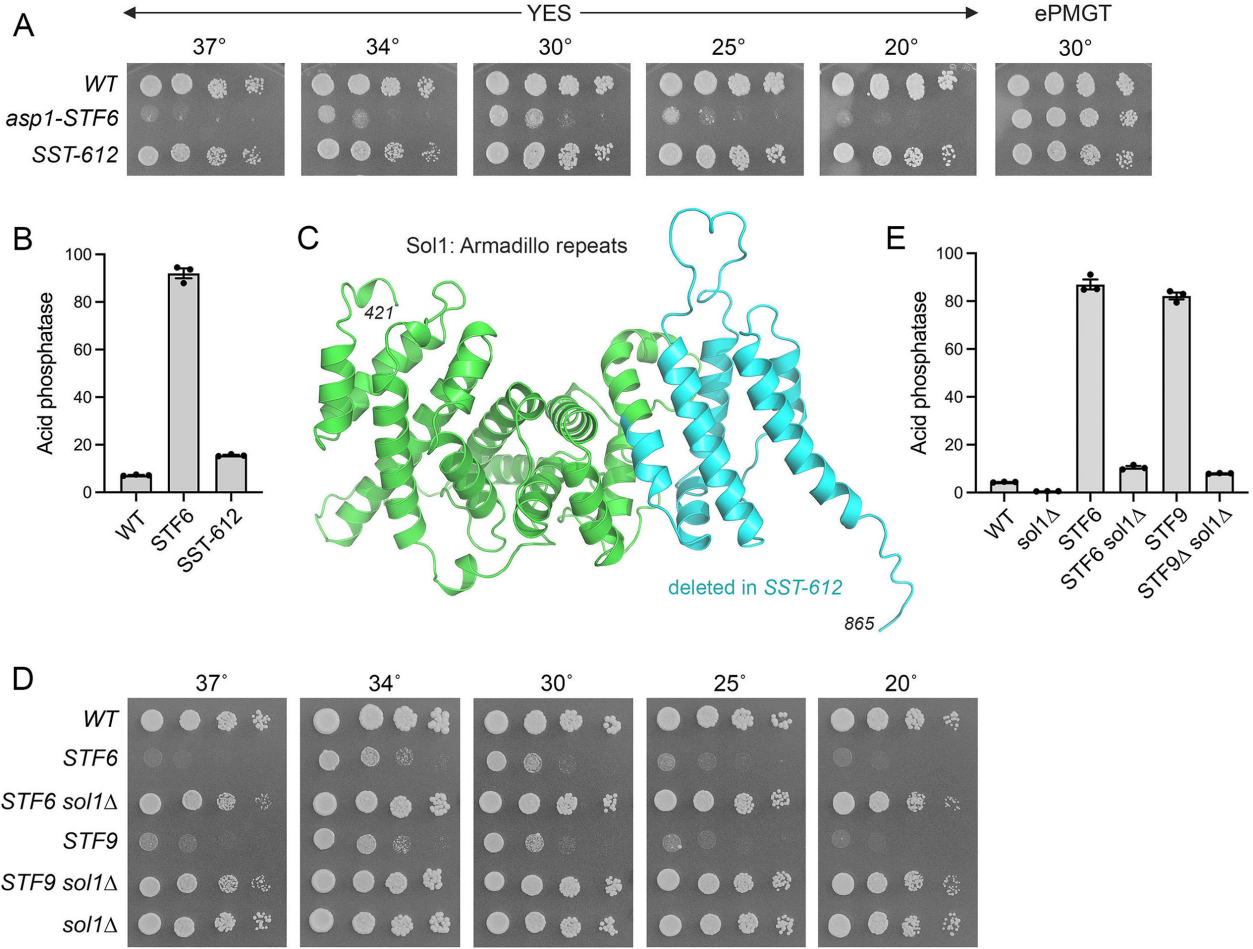
Isolation of a *sol1* mutant as a suppressor of IPP toxicosis. (**A**) Serial fivefold dilutions of wild-type, *asp1-STF6*, and *SST-612* (a spontaneous suppressor of *asp1-STF6*) cells were spot-tested for growth on YES agar at the indicated temperatures and on ePMGT agar at 30°C. (**B**) Wild-type, *asp1-STF6*, and *SST-612* cells were grown to *A*_600_ of 0.5 to 0.8 in liquid culture in ePMGT medium at 30°C, then harvested, washed, and assayed for acid phosphatase activity. (**C**) The AlphaFold model of the Sol1 C-terminal domain, consisting of tandem Armadillo repeats, is shown. A frameshift mutation in the *SST-612* strain results in the deletion of the C-terminal segment (aa 734–865) of the Sol protein (colored cyan). (**D**) Serial fivefold dilutions of fission yeast strains (as specified on the left) were spot-tested for growth on YES agar at the indicated temperatures. (**E**) The indicated strains grown in ePMGT at 30°C were assayed for acid phosphatase activity.

The ability to interrogate *asp1-STF6 and STF9* strains under permissive (ePMGT medium) versus restrictive (YES medium) conditions facilitated a screen for novel *SST* mutants by plating *STF6* or *STF9* cells on YES agar at 30°C and selecting rare single colonies that grew to large size against a background of tiny colonies (27). A new iteration of the screen for suppressors of *STF6* yielded *SST-612*, which restored growth on YES medium at 20–37°C vis-à-vis the severely sick growth phenotype of the parental *STF6* strain (Fig. 6A). The derepression of Pho1 expression characteristic of *STF6* cells was erased in the *SST-612* strain (Fig. 6B). Whole-genome sequencing of *SST-612* revealed two mutations: (i) a + 1 frameshift at the codon for amino acid 734 of the 865-aa Sol1 subunit of the SWI/SNF complex and (ii) a +1 frameshift at the codon for amino acid 1332 of the 1465-aa Abc3 vacuolar heme transporter. Our presumption (borne out below) was that the Sol1 mutation was responsible for the *SST* phenotype. Sol1 is the fission yeast counterpart of the budding yeast SWI/SNF subunit Swi1. As modeled in AlphaFold, the C-terminal half of Sol1 consists of tandem armadillo repeats (depicted in Fig. 6C) akin to the armadillo repeats observed in the cryo-EM structure of Swi1 (39). The Sol1 frameshift mutation in *SST-612* results in the translation of a foreign nonapeptide following Phe733 and subsequent truncation of Sol1 at a new stop codon, leading to deletion of the C-terminal segment of the armadillo repeat domain (colored cyan in Fig. 6C) and likely resulting in a loss of Sol1 function. Because *sol1* is a nonessential gene (28), we proceeded to construct a *sol1*∆ strain and then *sol1*∆ *asp1-STF6* and *sol1*∆ *asp1-STF9* double mutants. The salient finding was that *sol1*∆ recapitulated suppression of the *asp1-STF6/9* growth defect on the YES medium (Fig. 6D). *sol1*∆ reversed the *asp1-STF6/9* derepression of Pho1 and was by itself hyper-repressive of Pho1, by eightfold, in an *asp1*^+^ wild-type background (Fig. 6E, *P* value 0.0004). Thus, the *SST* suppressor screens “hit” two different subunits of the fission yeast SWI/SNF complex.

### Deletion of chromatin remodeling ATPase Swr1 does not suppress *asp1-STF* toxicosis

*S. pombe* has three additional multi-subunit chromatin remodeling machines—the SWR, INO80, and RSC complexes—anchored by the Snf2-family ATPases Swr1, Ino80, and Snf21, respectively. Swr1, like Snf22, is nonessential for fission yeast growth, whereas Ino80 and Snf21 are essential. The SWR complex, with its ATPase subunit Swr1 (1288-aa), mediates the incorporation of histone variant H2A.Z(Pht1) into nucleosomes at gene promoters (40, 41). To test whether the genetic suppression of IP_8_ toxicosis by *snf22*∆ was specific to the inactivation of the SWI/SNF complex, we asked whether a loss of SWR complex function could elicit equivalent suppression phenotypes. We found that deleting *swr1^+^* had no effect on fission yeast growth at 30°C on ePMGT or YES medium and that *STF6 swr1*∆ and *STF9 swr1*∆ double mutants grew well on ePMGT medium (Fig. S2A). However, unlike *snf22*∆, which suppressed the *STF6* and *STF9* growth defects on YES medium, *STF6 swr1*∆ and *STF9 swr1*∆ double mutants grew more poorly than the respective *STF6* and *STF9* single mutants when tested for growth on YES or ePMGT plus 250 µM GPC (Fig. S2A). We conclude that *asp1-STF* toxicosis is specifically dependent on the SWI/SNF chromatin remodeling complex. We also found that *swr1*∆ *snf22*∆ and *swr1*∆ *snf22-*(*D996A-E997A*) double mutants grew well on YES agar at 20–37°C (Fig. S2B), signifying that these two chromatin remodelers are not functionally redundant for sustaining vegetative growth.

### RNA-seq profiling of *snf22*∆ and *snf22-*(*D996A-E997A*) cells

Monahan et al. (28) employed microarray analysis to compare the transcriptional profiles of wild-type and *snf22*∆ fission yeast strains and reported that 53 mRNAs were upregulated by twofold or greater and 54 mRNAs were downregulated by twofold or greater. Here, we performed RNA-seq in parallel on poly(A)^+^ RNA isolated from *snf22*∆, *snf22-*(*D996A-E997A*), and wild-type *snf22*^+^ cells. cDNAs obtained from three biological replicates (using RNA from cells grown to mid-log phase in YES medium at 30°C) were sequenced for each strain. 93% to 97% of the sequence reads (19 to 27 million per replicate) were aligned to genomic loci (Table S1). Read densities for individual genes were highly reproducible between biological replicates (Pearson coefficients of 0.978 to 0.988) (Table S2). A cutoff of ±2-fold change in normalized transcript read level and an adjusted *P*-value of ≤0.05 were the criteria applied to derive an initial list of differentially expressed annotated loci in the *snf22* mutant versus the wild-type control. We then focused on differentially expressed genes with average normalized read counts ≥100 in either the mutant or wild-type strain to eliminate transcripts that were expressed at very low levels in vegetative cells. We thereby identified sets of 94 and 117 annotated protein-coding genes that were, respectively, upregulated and downregulated by these criteria in *snf22*∆ cells (Table S3) and sets of 73 and 87 protein-coding genes that were, respectively, upregulated and downregulated in *snf22-*(*D996A-E997A*) cells (Table S4). The genes dysregulated by the absence of Snf22 and by inactivation of its ATPase were highly correlated: to wit, 42 transcripts were upregulated in both mutants (*P* < 7.3 e^−58^) and 67 transcripts were downregulated in both mutants (*P* < 1.2 e^−101^) (Table S5).

**Fig 7:**
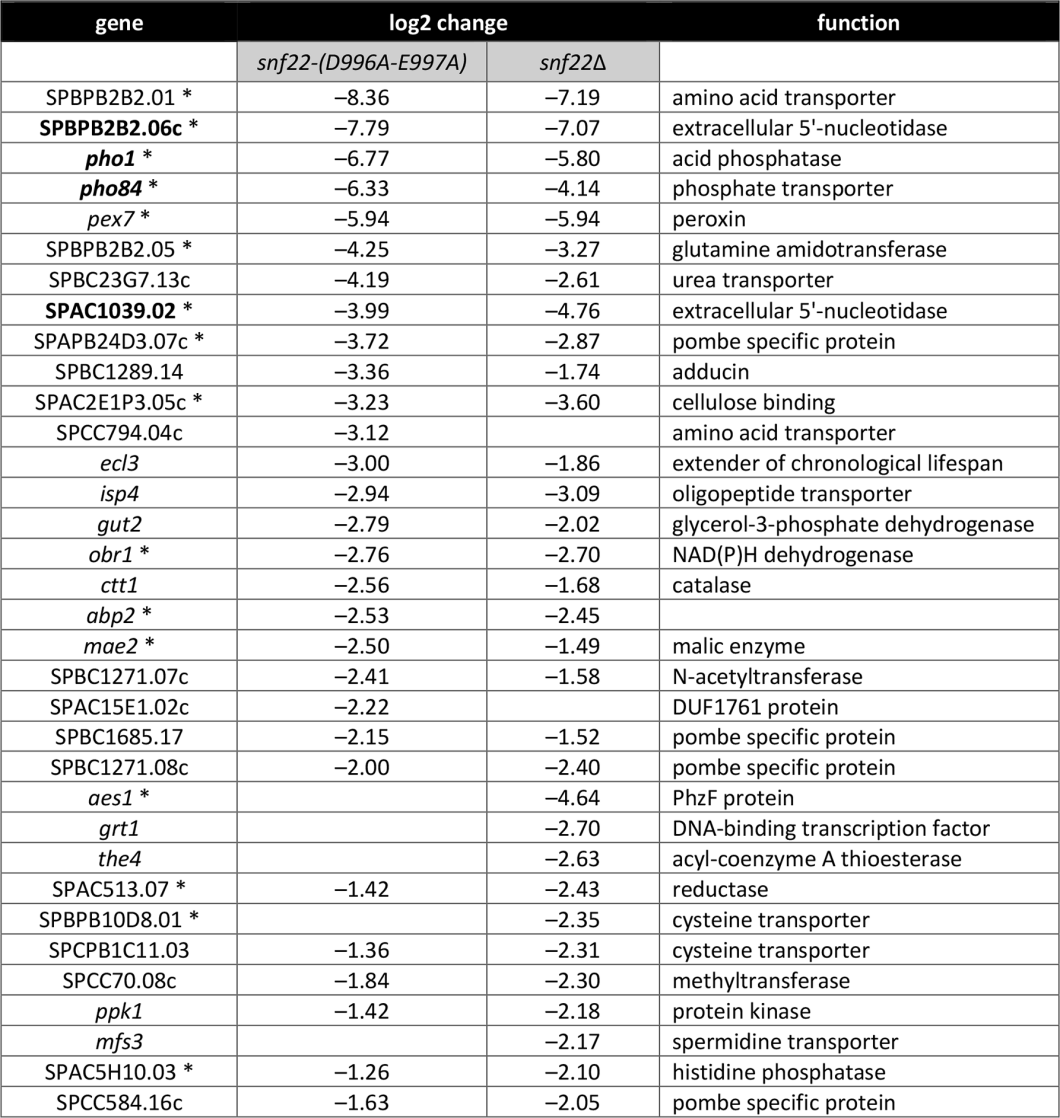
RNA-seq defines a Snf22-dependent gene set. List of the coding genes that were downregulated by at least fourfold (log2 change of at least –2.0) in *snf22-(D996A-E997A*) cells (*n* = 23) and *snf22*∆ cells (*n* = 26), of which 27 genes were coordinately down by at least twofold (log change of at least –1.0) in both strains. Sixteen of the most strongly downregulated transcripts that had been detected by previous microarray analysis of *snf22*∆ are denoted by asterisks.

Figure 7 shows the coding genes that were downregulated by at least fourfold (log2 change of at least –2.0) in *snf22-*(*D996A-E997A*) cells (*n* = 23) and *snf22*∆ cells (*n* = 26), of which 27 genes were coordinately down by at least twofold (log2 change of at least –1.0) in both strains. Sixteen of the most strongly downregulated transcripts had been detected by microarray analysis of *snf22*∆ (denoted by asterisks in Fig. 7), though the magnitude of the downregulation was typically less for the microarray data compared to RNA-seq. Genes involved in phosphate acquisition were strongly hyper-repressed: (i) *pho1* by 109-fold and 56-fold in *snf22-*(*D996A-E997A*) cells and *snf22*∆ cells, respectively; (ii) *pho84* by 80-fold and 18-fold, respectively; (iii) extracellular 5′-nucleotidase *SPBPB2B2.06c* by 220-fold and 134-fold, respectively; and (iv) extracellular 5′-nucleotidase *SPAC1039.02* by 16-fold and 27-fold, respectively (Fig. 7). *tgp1*, the third gene in the fission yeast *PHO* operon, is expressed in wild-type cells at a much lower level (average normalized read count of 669) than either *pho1* (read count of 35,589) or *pho84* (read count of 28,610). *tgp1* expression was downregulated by 2.5-fold in *snf22-*(*D996A-E997A*) cells (Table S4). Expression of the *pho7* gene, encoding the Pho7 transcription factor that drives the *PHO* regulon, was unaffected by the *snf22* mutations.

It is noteworthy that the *ecl3* gene located immediately upstream of the tandem *pho84–pho1* locus was downregulated by 8-fold and 3.6-fold in *snf22-*(*D996A-E997A*) cells and *snf22*∆ cells, respectively. Up and down changes in *ecl3* expression mirror those of *pho1* in multiple other mutant backgrounds that affect phosphate homeostasis (12, 15, 31). Moreover, several genes located just downstream of the tandem *pho84–pho1* locus were also downregulated in *snf22-*(*D996A-E997A*) cells and *snf22*∆ cells, including (i) *SPBPB2B2.01*, by 329-fold and 146-fold, respectively; (ii) *SPBPB2B2.05*, by 19-fold and 10-fold, respectively; and (iii) *SPBPB2B2.06c*, as cited above. These downregulated genes are located within a 30-kbp segment of chromosome II that embraces *pho84* and *pho1*.

Figure S3 compiles the coding genes that were upregulated by at least fourfold (log2 change of at least 2.0) in *snf22-*(*D996A-E997A*) cells (*n* = 17) or *snf22*∆ cells (*n* = 21), of which 18 genes were coordinately up by at least twofold (log2 change of at least 1.0) in both strains. Fourteen of the most strongly upregulated transcripts observed by RNA-seq had been detected by microarray analysis of *snf22*∆ (denoted by asterisks in Fig. S3). Several genes of the fission yeast iron homeostasis regulon (42, 43) were derepressed in *snf22-*(*D996A-E997A*) cells, including *str3* (up 87-fold;) *frp1* (up 4.6-fold); *fio1* (up 4.5-fold); *str1* (up 4-fold); *fip1* (up 3.3-fold); *abc3* (up 3.2-fold); and *srx1* (up 2-fold) (Fig. S3; Table S4). Expression of the *fep1* gene, encoding the Fep1 transcriptional repressor of the iron regulon, was unaffected by the *snf22* mutations. Genes for several sugar transporters and sugar metabolizing enzymes were also significantly upregulated in *snf22-*(*D996A-E997A*) and/or *snf22*∆ cells (Fig. S3). Our findings that iron and sugar utilization genes are subject to repressive control by Snf22 accord with those of Monahan et al. (28).

### *snf22-*(*D996A-E997A*) is not synthetically lethal or sick with *PHO* hyper-repressive 3′-processing and termination mutations

The results presented above show that loss of Snf22 protein or inactivation of its ATPase hyper-represses *pho1* and reverses the *pho1* derepression elicited by a variety of genetic mutations that favor precocious lncRNA 3′-processing/termination in response to RNA signals upstream of the *pho1* mRNA promoter. Because previous studies established that Pho1 derepression in such genetic backgrounds depends on CPF subunits Ssu72, Swd22, and Ppn1, termination factor Rhn1, the Thr4 “letter” of the CTD code, Asp1 kinase, and Spx1 (mutations of which are themselves *pho1* hyper-repressive), our initial suspicions were that Snf22 inactivation overrode the Pho1 derepressive mutations by eliminating or reducing precocious lncRNA termination. A key feature of the genetic landscape of the *pho1* hyper-repressive loss-of-function alleles is that they display a network of pairwise synthetic lethalities (e.g., *rpb1-T4A* is lethal with *ppn1*∆ and *swd22*∆; *ppn1*∆ and *swd22*∆ are lethal with *rhn1*∆ and *ssu72-C13S; asp1*∆ and *asp1-D333A* are lethal with *ppn1*∆, *swd22*∆, and *ssu72-C13S*), indicating that the ostensibly inessential CPF subunits, Rhn1, Asp1 kinase, and CTD-Thr4 function as important, albeit genetically redundant, agonists of 3′-processing/termination. If the same is true of Snf22, then we would expect to observe lethality or an overt growth defect when the *snf22-*(*D996A-E997A*) mutant is mated to CPF/Rhn1, Asp1 kinase, Spx1, and Rpb1-T4A mutants. To the contrary, after crossing differentially marked mutants and analysis of haploid progeny, we readily obtained viable double mutants combining *snf22-*(*D996A-E997A*) with *rpb1-T4A*, *rhn1*∆, *spx1*∆, CPF subunit mutants *ssu72-C13S*, *ppn1*∆ and *swd22*∆, and IPP kinase mutants *asp1*∆ and *asp1-D333A*. The *snf22-*(*D996A-E997A*) double mutants were spot-tested for growth on YES agar in parallel with the respective single mutants (Fig. S4). The *snf22-*(*D996A-E997A*) allele did not cause overt synthetic growth defects relative to the single mutants. In the cases of *swd22*∆, *asp1*∆, and *asp1-D333A*, which display a slow-growth phenotype at 20°C, the cold sensitivity was partially suppressed by *snf22-*(*D996A-E997A*). This absence of synthetic lethality or sickness suggests that the inactivation of Snf22 does not exert its effects via 3′-processing/termination.

### Effect of *snf22*∆ and *snf22-*(*D996A-E997A*) on *tgp1* promoter and *nctgp1* promoter activity

**Fig 8:**
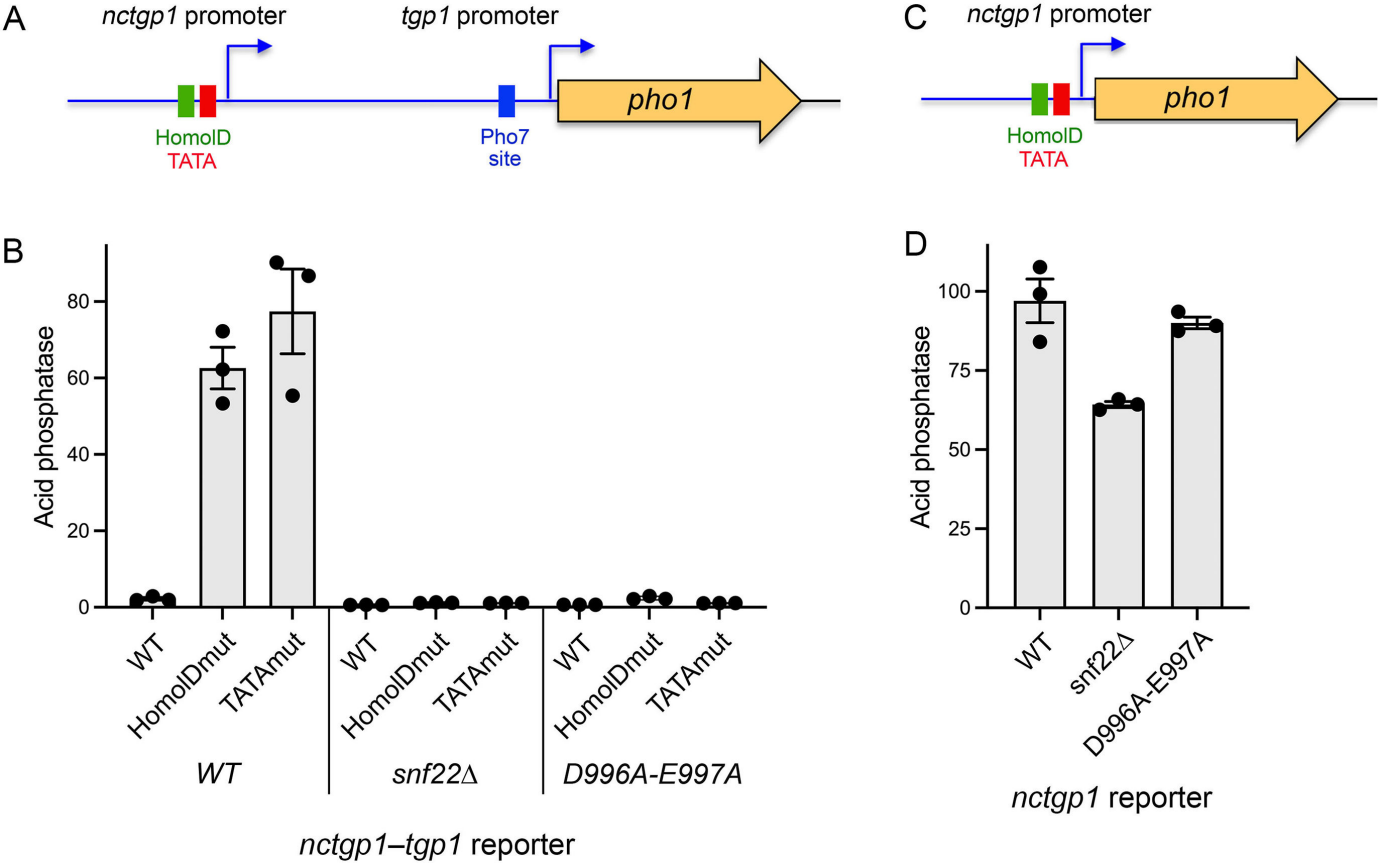
Effect of *snf22*∆ and *snf22-*(*D996A-E997A*) on *tgp1* promoter and *nctgp1* promoter activity. (**A**) Schematic of the plasmid-borne *nctgp1–tgp1_pro_·pho1* reporter in which *pho1* expression driven by the *tgp1* promoter is repressed by *nctgp1* lncRNA transcription. The HomolD and TATA boxes that comprise the *nctgp1* lncRNA promoter are depicted as green and red boxes, respectively. A single binding site for transcription factor Pho7 that drives the *tgp1* promoter is shown as a blue box. (**B**) *nctgp1–tgp1_pro_·pho1* reporter plasmids with a wild-type *nctgp1* promoter or with mutated versions in which the *nctgp1* promoter was inactivated by nucleotide substitutions in either the HomolD box or the TATA box (14) were transfected into wild-type, *snf22∆*, or *snf22-*(*D996A-E997A*) strains in which the chromosomal *pho1* locus was deleted. Transformants were selected and single colonies of individual transformants were pooled (>20) and grown in a plasmid-selective liquid medium to *A*_600_ of 0.5–0.8. Aliquots were harvested for acid phosphatase activity measurements. Each datum in the bar graph is the average of assays using cells from three independent cultures ± SEM. (**C**) A reporter of *nctgp1* promoter activity in which the *nctgp1* promoter directly drives transcription of the *pho1* gene. (**D**) The *nctgp1·pho1* reporter plasmid was transfected into wild-type, *snf22∆*, or *snf22-*(*D996A-E997A*) strains in which the chromosomal *pho1* locus was deleted. Pools of individual transformants were assayed for acid phosphatase activity as in panel B.

To address these issues, we employed a plasmid-borne *nctgp1–tgp1_pro_·pho1* reporter, in which a *pho1* open-reading frame (ORF) under the control of the *tgp1* promoter replaces the *tgp1* ORF (Fig. 8A). Introduction of this reporter into fission yeast cells in which the chromosomal *pho1* gene is deleted allows for quantification of *tgp1* promoter activity via measurement of Pho1 acid phosphatase expression. This reporter recapitulates known homeostatic controls on the native *nctgp1–tgp1* locus (14). The *nctgp1* lncRNA promoter has a distinctive bipartite architecture, consisting of a TATA-box (TATATATA) situated 22 nucleotides upstream of the *nctgp1* transcription start site and a HomolD-box (CAGTCACA) located 26 nucleotides upstream of the TATA-box (Fig. 8A) (14). A similar bipartite HomolD/TATA promoter structure is characteristic of the *prt2* and *prt* lncRNA genes that interfere with *pho84* and *pho1* mRNA expression (8). The *tgp1* promoter consists of a TATA box (TATTTAA) 28 nucleotides upstream of the *tgp1* transcription start site and a Pho7 binding site (TCGGACATTCAA) located 180 nucleotides upstream of the *tgp1* start site (9, 10). Nucleotide substitutions in either the HomolD box or the TATA-box inactivate the *nctgp1* lncRNA promoter and result in derepression of the otherwise repressed flanking *tgp1* promoter, as gauged by acid phosphatase activity (Fig. 8B) (14). These HomolD/TATA lncRNA promoter mutant *nctgp1-tgp1_pro_-pho1* reporters thereby provide a readout of the strength of the *tgp1* promoter driving the *pho1* ORF in the absence of any lncRNA interference. The instructive finding was that the derepressed expression of the *nctgp1* HomolD/TATA mutant *tgp1*_pro_-*pho1* reporter was greatly reduced in *snf22*∆ and *snf22-*(*D996A-E997A*) cells (Fig. 8B), signifying that Snf22 and its ATPase are needed for *tgp1* promoter function.

To gauge the *nctgp1* promoter *per se*, we deployed a reporter plasmid in which the *nctgp1* promoter directly drives the *pho1* ORF (Fig. 8C) (14). As noted previously (8), the strength of the *nctgp1* promoter, as gauged by Pho1 acid phosphatase expression, is slightly greater than that of the *tgp1* promoter, as might be expected for a mechanism of lncRNA-mediated transcriptional interference with the downstream mRNA gene. The relevant finding here is that the activity of the *nctgp1* promoter was reduced only modestly in *snf22*∆ cells (by one-third; *P* value 0.04) and insignificantly in *snf22-*(*D996A-E997A*) cells (*P* value 0.43) (Fig. 8D). We surmise that interdicting Snf22 function leads to suppression of IP_8_ toxicosis on YES medium by blunting the derepression of the *tgp1* mRNA promoter in the various *asp1-STF* strains and thereby the uptake of GPC in the medium that elicits the *asp1-STF* growth defect.

### Effect of *snf22*∆ and *snf22-*(*D996A-E997A*) on *prt–pho1* expression and *prt* promoter activity

**Fig 9:**
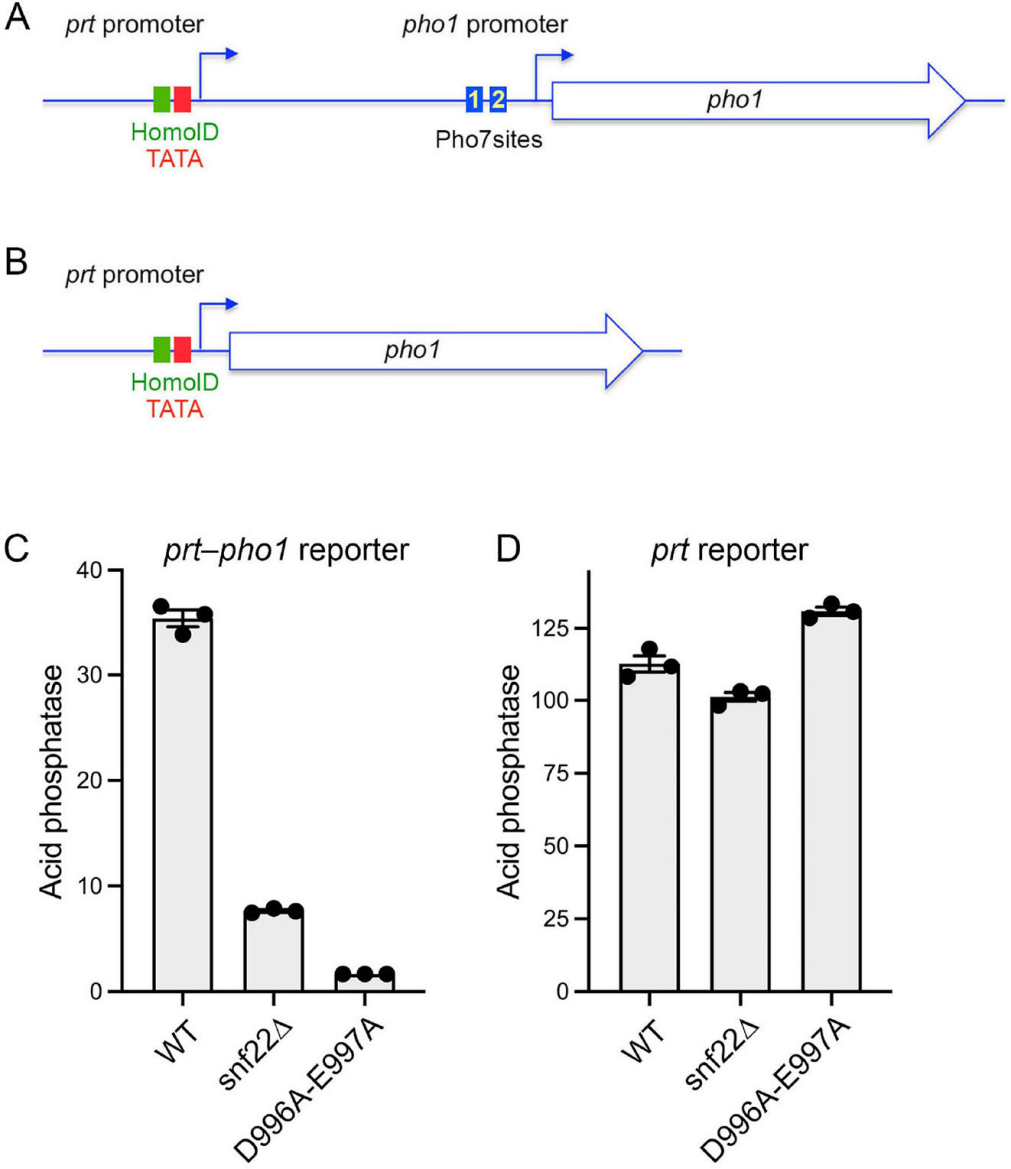
Effect of *snf22*∆ and *snf22-(D996A-E997A*) on *prt–pho1* expression and *prt* promoter activity. (**A**) Schematic of the plasmid-borne *prt–pho1* reporter in which *pho1* expression is repressed by *prt* lncRNA transcription. The HomolD and TATA boxes that comprise the *prt* lncRNA promoter are depicted as green and red boxes, respectively. Two binding sites for transcription factor Pho7 that drives *pho1* mRNA transcription are shown as blue boxes. (**B**) A reporter of *prt* promoter activity in which the *prt* promoter directly drives transcription of the *pho1* gene. (**C and D**) The indicated reporter plasmids were transfected into wild-type, *snf22*∆, or *snf22-*(*D996A-E997A*) strains in which the chromosomal *pho1* locus was deleted. Transformants were selected and single colonies of individual transformants were pooled (>20) and grown in plasmid-selective liquid medium to *A*_600_ of 0.5–0.8. Aliquots were harvested for acid phosphatase activity measurements. Each datum in the bar graph is the average of assays using cells from three independent cultures ± SEM.

Pho1 expression from a plasmid-borne *prt–pho1* locus faithfully responds to phosphate starvation and various genetic mutations that derepress or hyper-repress the chromosomal *pho1* gene (15, 31, 44). The *prt* lncRNA promoter consists of HomolD and TATA boxes preceding the lncRNA transcription start site (Fig. 9A). The *pho1* promoter consists of a TATA box (TATTTAA) 27 nucleotides upstream of the *pho1* transcription start site as well as two Pho7 binding sites—site 1 (TCGCTGCTTGAA) and site 2 (TCGGAAATTAAA)—located further upstream, in direct repeat orientation and separated by a 20-nt spacer (9, 11) (Fig. 9A). The basal level of Pho1 expression from the *prt–pho1* reporter plasmid in *snf22*^+^ wild-type cells was reduced by 5-fold and 21-fold in *snf22*∆ and *snf22-*(*D996A-E997A*) cells, respectively (Fig. 9C), consistent with the relative impact of these *snf22* alleles on the expression of *pho1* mRNA and Pho1 acid phosphatase activity from the chromosomal *prt–pho1* locus, whereby the ATPase mutation had a stronger effect than the gene deletion. To assess the *prt* promoter, we used a *prt·pho1* reporter plasmid in which the *prt* promoter directly drives the *pho1* ORF (Fig. 9B) (44). The salient findings were that *snf22*∆ had little effect on *prt* promoter activity whereas *snf22-*(*D996A-E997A*) resulted in a slight (16%) increase in activity versus *snf22*^+^ (Fig. 9D). These results indicate that the reduced *pho1* mRNA expression in *snf22* mutants reflects diminished activity of the mRNA promoter rather than the increased activity of the lncRNA promoter.

### ***snf22*****∆ and** ***snf22-*****(*****D996A-E997A*****) block the increase in** ***pho1*** **mRNA in** ***asp1-H397A*****,** ***seb1-G476S*****, and** ***rad24*****∆ cells**

**Fig 10:**
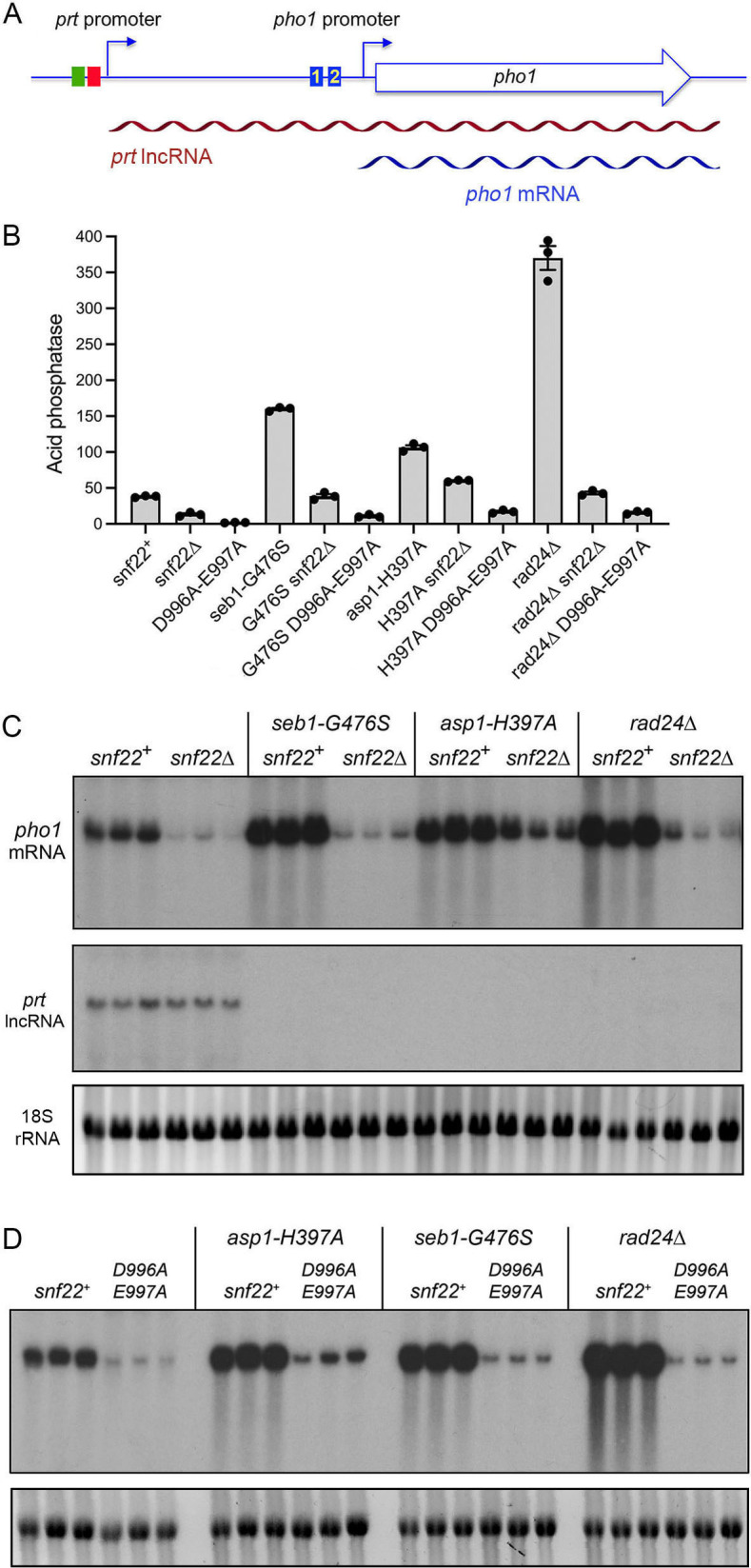
*snf22*∆ and *snf22-(D996A-E997A*) block the increase in *pho1* mRNA in *seb1-G476S*, *asp1-H397A*, and *rad24*∆ cells. (**A**) Schematic of the plasmid-borne *prt–pho1* reporter highlighting the *pho1* mRNA (blue) and the interfering *prt* lncRNA (red) that traverses the *pho1* promoter and terminates at the *pho1* mRNA poly(A) site. (**B**) The *prt–pho1* reporter plasmid was transfected into *pho1∆* cells with the genotypes specified on the *x*-axis. Pools of individual transformants were assayed for acid phosphatase activity. The Pho1-derepression seen in *seb1-G476S*, *asp1-H397A*, and *rad24*∆ mutants was ameliorated or abolished in the *snf22*∆ and *snf22-*(*D996A-E997A*) genetic backgrounds. (**C**) RNA was isolated from three independent cultures of *pho1*∆ cells bearing the *prt-pho1* reporter plasmid; the genotypes are indicated above the panels. The RNAs were resolved by formaldehyde-agarose gel electrophoresis. The gel was photographed under UV light to visualize the ribosomal RNAs (the 18S rRNA is shown in the bottom panel) to establish a sample loading control. The RNAs were then transferred to a membrane and hybridized to a *pho1* probe (top panel) or a *prt* probe (middle panel). (**D**) RNA isolated from three independent cultures of *pho1∆* cells (either *snf22^+^* wild-type or *D996A-E997A* as specified) bearing the *prt-pho1* reporter plasmid was analyzed by Northern blotting using a radiolabeled probe complementary to the *pho1* mRNA (top panel). The 18S rRNA loading controls are shown in the bottom panel.

Introducing the *prt–pho1* reporter into *pho1*-deleted *asp1-H397A*, *seb1-G476S*, and *rad24*∆ strains led to derepression of Pho1 acid phosphatase activity *vis-à-vis* the wild-type *snf22*^+^ control strain (Fig. 10B). As is the case when Pho1 is expressed from the chromosomal *prt–pho1* locus, the extent of derepression of the plasmid Pho1 reporter acid phosphatase activity was greatest in the *rad24*∆ strain (Fig. 10B) (31). When the *prt–pho1* reporter was introduced into *asp1-H397A*, *seb1-G476S*, and *rad24*∆ strains bearing *snf22*∆ or *snf22-*(*D996A-E997A*) mutations, the derepression of Pho1 was blunted or effaced, with the ATPase mutation again exerting a stronger effect than the gene deletion (Fig. 10B). Having affirmed that the reporter system is responsive to *pho1*-derepressing mutations at the protein levels, we performed Northern analysis on total RNA isolated from reporter plasmid-bearing *asp1-H397A*, *seb1-G476S*, and *rad24*∆ strains with either *snf22*^+^ wild-type or *snf22*∆ alleles. RNAs from three biological replicate cultures were resolved in parallel by agarose gel electrophoresis and transferred to a membrane. A 5’ ^32^P-labeled ssDNA complementary to nucleotides 84–115 downstream of the *pho1* transcription start site annealed to the ~1.6 kb *pho1* mRNA (Fig. 10C) that extends from the transcription start site 51 nucleotides 5′ of the translation start site to a poly(A) site 135 nucleotides 3′ of the stop codon (45, 46) (Fig. 10A). Compared to the *snf22*^+^ control, the abundance of the *pho1* mRNA was increased in *asp1-H397A*, *seb1-G476S*, and *rad24*∆ cells (Fig. 10C), consistent with the derepression of Pho1 acid phosphatase activity. The effect of *snf22*∆ was to decrease the basal level of the *pho1* mRNA in otherwise wild-type cells (consistent with the RT-qPCR and RNA-seq results) and to lessen (in *asp1-H397A* cells) or suppress (in *seb1-G476S* and *rad24*∆ cells) the increase in *pho1* mRNA (Fig. 10C). Figure 10D shows a Northern analysis of *pho1* mRNA derived from the *prt–pho1* reporter plasmid in *snf22*^+^ versus *snf22-*(*D996A-E997A*) cells. The ATPase-defective Snf22 mutant dampened the *pho1* mRNA in otherwise wild-type cells and blocked the increase in *pho1* mRNA in the *asp1-H397A*, *seb1-G476S*, and *rad24*∆ mutants.

The use of the multicopy *prt–pho1* reporter plasmid is advantageous for the detection of the *prt* lncRNA, which is rapidly degraded by the nuclear exosome (32, 45). Probing the Northern blot with a ^32^P-labeled ssDNA complementary to the segment of the *prt* RNA from nucleotides +160 to +202 revealed a ~2.5 kb RNA corresponding to a *prt–pho1* read-through transcript, this being the lncRNA that interferes with *pho1* mRNA synthesis (Fig. 10A and C). The steady-state level of the *prt* lncRNA was apparently the same in *snf22*^+^ and *snf22*∆ cells (Fig. 10C). As noted previously, the *prt–pho1* read-through transcript was absent from reporter plasmid-bearing *asp1-H397A*, *seb1-G476S*, and *rad24*∆ strains, in which transcription of the lncRNA is terminated precociously in response to *cis*-acting PAS and DSR elements in the nascent *prt* transcript (15, 24, 31). The derepression of *pho1* in *asp1-H397A*, *seb1-G476S*, and *rad24*∆ cells can be reversed by mutating the PAS and DSR elements, which overrides precocious termination and restores synthesis of the *prt–pho1* read-through lncRNA (15, 24, 31). The key observation here is that the absence of the *prt–pho1* read-through transcript in *asp1-H397A*, *seb1-G476S*, and *rad24*∆ cells persists when *snf22* is ablated (Fig. 10C). Thus, it is not the case that interdiction by *snf22*∆ of the derepression of *pho1* is caused by restitution of full-length interfering *prt* lncRNA production.

### Pho7 binding site mutations enhance the Snf22 dependence of *pho1* promoter activity

**Fig 11:**
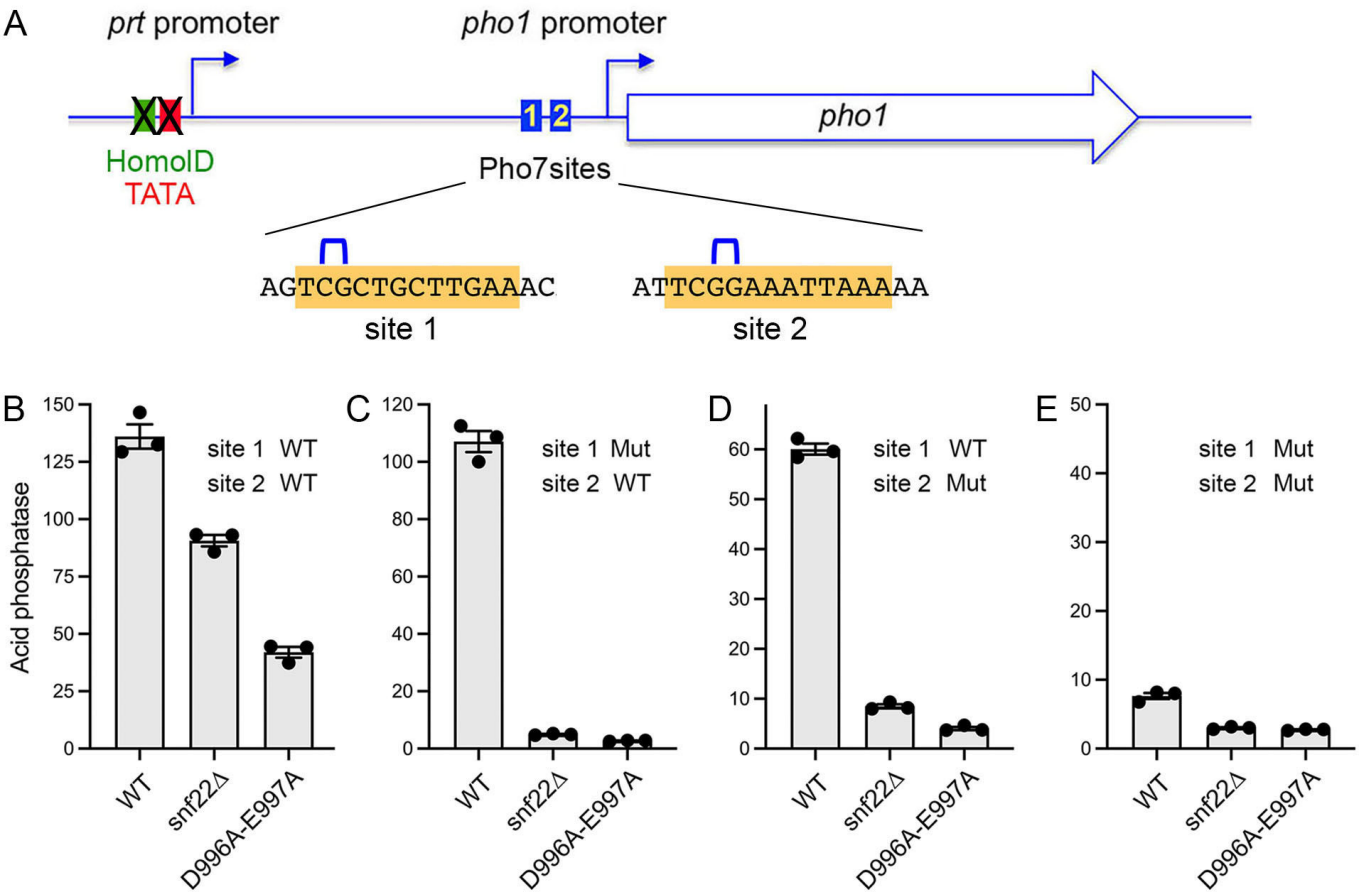
Pho7 binding site mutations enhance the Snf22 dependence of Pho1 expression. (**A**) Schematic of the plasmid-borne reporter of *pho1* promoter activity in which *prt* lncRNA transcription is abolished by mutations (indicated by X) in the HomolD and TATA box elements in the *prt* promoter. Pho7 binding sites 1 and 2 in the *pho1* promoter are depicted as blue boxes and the nucleotide sequence of each site is highlighted in gold. Mutations in each Pho7 binding site are indicated by blue brackets above the sequences. (**B–E**) The indicated reporter plasmids were transfected into *snf22^+^* (WT), *snf22*∆, or *snf22-*(*D996A-E997A*) strains in which the chromosomal *pho1* locus was deleted. Pools of individual transformants were assayed for acid phosphatase activity. Each datum in the bar graph is the average of assays using cells from three independent cultures ± SEM.

The near total blockade of derepressed *tgp1* promoter activity by *snf22*∆ and *snf22-*(*D996A-E997A*) when the *nctgp1* lncRNA was shut off by HomolD/TATA mutations (Fig. 8B) stood in contrast to the partial reductions in de-repressed *pho1* promoter activity in *snf22*∆ and *snf22-*(*D996A-E997A*) cells (to 66% and 31%, respectively, of the *snf22*^+^ wild-type control) when the interfering *prt* promoter was shut off (Fig. 11B). We considered the possibility that the differences in Snf22 dependence might be related to the fact that the *tgp1* promoter contains one Pho7 binding site, whereas the *pho1* promoter contains two Pho7 binding sites. To address this hypothesis, we made dinucleotide deletions of essential bases within sites 1 and 2 of the *pho1* promoter, singly and in combination, in the context of the HomolD/TATA-mutant *prt–pho1* reporter (Fig. 11A). Simultaneous mutations of both Pho7 sites reduced Pho1 reporter activity in *snf22*^+^ cells to 6% of the unmutated control reporter (Fig. 11E), an effect consistent with the impact of mutating essential amino acids of the Pho7 DNA-binding domain (9). Whereas singly mutating Pho7 binding site 1 had only a mild effect on reporter expression in *snf22*^+^ cells (79% of the unmutated control reporter), the site 1 mutant was now acutely dependent on Snf22 for Pho1 expression. To wit, site 1 mutant reporter activities in *snf22*∆ and *snf22-*(*D996A-E997A*) cells were 5% and 3%, respectively, of the *snf22*^+^ wild-type control (Fig. 11C). Singly mutating Pho7 site 2 reduced reporter expression to 44% of the unmutated control in *snf22*^+^ cells and also enhanced its Snf22-dependence, insofar as site 2 reporter activity in *snf22*∆ and *snf22-*(*D996A-E997A*) cells was 14% and 7%, respectively, of the *snf22*^+^ wild-type control (Fig. 11D). The more modest contribution of site 1 versus site 2 to *pho1* expression from the reporter plasmid resonates with prior findings that the affinity of the Pho7 DNA binding domain is threefold greater *in vitro* for site 2 than for site 1 (11).

### ***snf22*** **∆ delays the onset of the phosphate starvation response**

**Fig 12:**
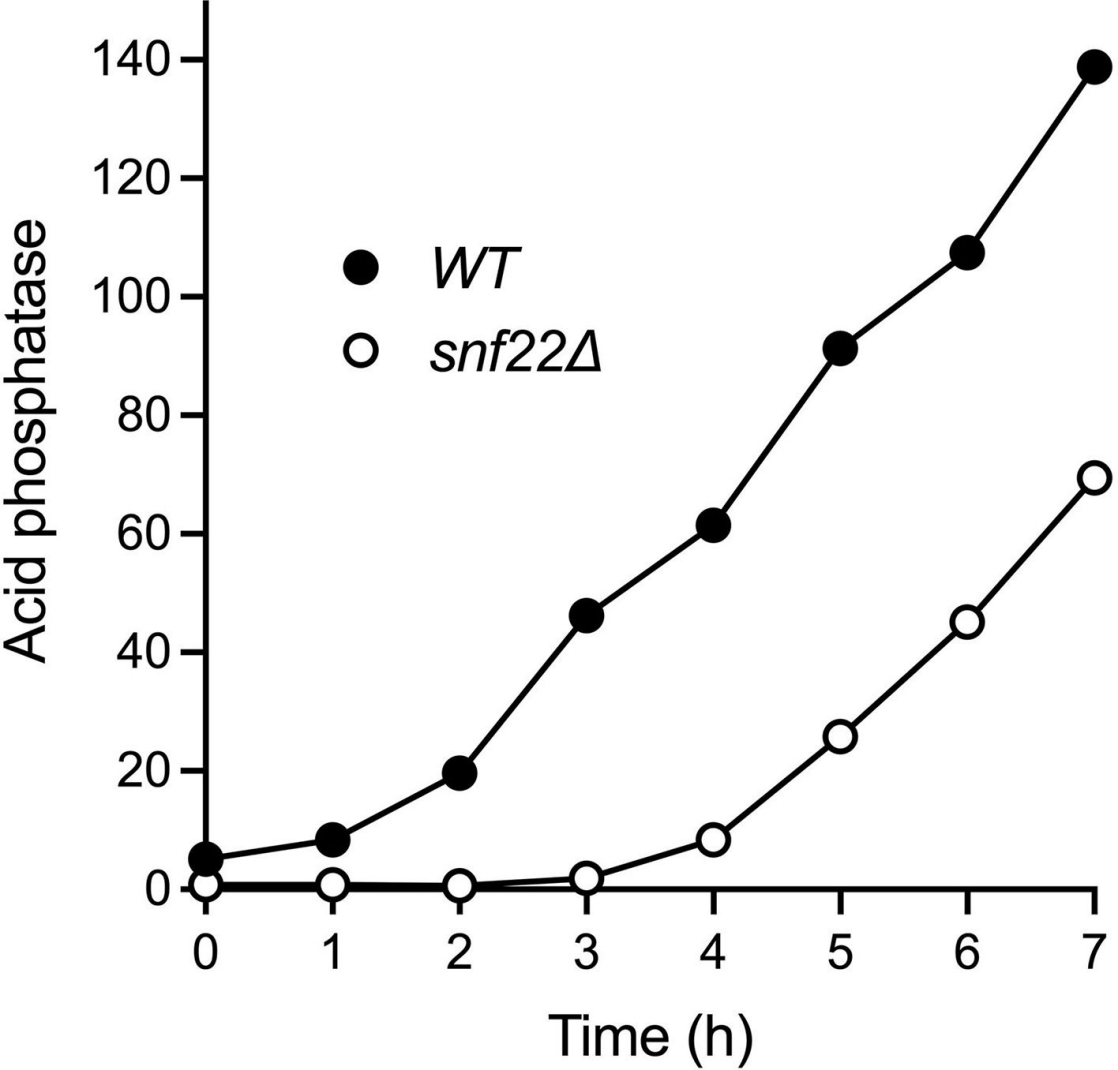
*snf22*∆ delays the onset of the phosphate starvation response. Wild-type and *snf22*∆ cells were assayed for Pho1 acid phosphatase activity prior to (time 0) and at hourly intervals after transfer to a ePMGT medium lacking phosphate. Data are the average of three independent experiments ± SEM. The error values are small such that the error bars do not extend beyond the symbols.

Wild-type and *snf22*∆ cells were grown in liquid culture in YES medium, washed with water, and then incubated in synthetic ePMGT medium lacking exogenous phosphate. Aliquots of the cultures were assayed for Pho1 acid phosphatase activity prior to and at hourly intervals after the transfer to a phosphate-free medium. Wild-type cells respond to phosphate starvation by derepressing *pho1* transcription and thereby steadily accumulating Pho1 enzyme during the interval from 1 to 7 h post-starvation. *snf22*∆ cells, which have a lower basal level of Pho1 expression, experienced a prolonged lag phase prior to the onset of Pho1 accumulation, after which activity increased with virtually the same slope as seen in wild-type cells (Fig. 12). Comparison of the two kinetic profiles indicates that *snf22*∆ elicited a 3-h delay in the phosphate starvation response.

## DISCUSSION

Repression of the fission yeast *PHO* regulon by lncRNA-mediated transcriptional interference is sensitive to genetic manipulations of inositol pyrophosphate dynamics. Pyrophosphatase mutations leading to increased intracellular IP_8_ derepress the *PHO* genes via the action of IP_8_ as an agonist of precocious lncRNA 3′-processing/termination. *asp1-STF* mutations resulting in too much IP_8_ are cytotoxic in YES medium owing to overexpression of the Tgp1 glycerophosphodiester transporter (27). Previous epistasis tests and genetic screens for suppression of *asp1-STF* growth defects had highlighted three means by which IP_8_ toxicosis can be circumvented: (i) by mutations of components of transcription and the 3′-processing/termination machinery that dampen the impact of toxic IP_8_ levels on Pol2 termination; (ii) by mutations of proteins Spx1, Gde1, and Vtc4, each of which contains an SPX domain that acts as an inositol pyrophosphate sensor; and (iii) by mutations in two upstream enzymes of inositol polyphosphate metabolism: the phospholipase C enzyme Plc1 that generates IP_3_; and the essential Kcs1 kinase that converts IP_6_ to 5-IP_7_, the immediate precursor of IP_8_.

Here we identified the Snf22 ATPase subunit and the Sol1 subunit of the fission yeast SWI/SNF chromatin remodeling complex as critical abettors of IP_8_ toxicosis. The *snf22* allele isolated in the screen for suppression of *asp1-STF7* toxicity has a frameshift mutation which eliminates a C-terminal Bromo domain that binds acetyllysine-modified histones (29). Subsequent analyses employing *snf22*∆ null and ATPase-defective *snf22-*(*D996A-E997A*) alleles established that the evasion of IP_8_ toxicosis is via a novel route *vis-à-vis* previously characterized transcription-related suppressors that counteract precocious lncRNA termination. We find that Snf22 is required for the derepressed transcription of the *tgp1* mRNA in *asp1-STF* cells (Fig. 5) and the activity of the derepressed *tgp1* promoter in the plasmid reporter system (Fig. 8B).

Derepression of *tgp1* above a threshold level appears to be the determinant of IP_8_ toxicity on YES medium, insofar as RNA-seq analyses show that *tgp1* mRNA levels are increased by 43-fold and 47-fold in *asp1-STF6* and *asp1-STF9* mutants that manifest toxicity versus only 21-fold and 7-fold *tgp1* mRNA increases in *asp1-H397A* cells and *aps1*∆ cells that grow normally on YES medium. Sub-threshold levels of *tgp1* derepression that do not result in toxicity on YES medium are observed in *rad24*∆ (25-fold increase), *CTD-S5-S5A* and *CTD-P6-P6A* (23-fold increase), and *seb1-G476S* (9-fold increase) mutant strains (22, 24, 31). (Note that the present RT-qPCR-based assay returned a 17-fold increase in *tgp1* mRNA in *asp1-STF6* cells; we regard RNA-seq as a more accurate gauge of *tgp1* mRNA levels.)

Snf22 is also required for the derepression of *pho1* in nontoxic genetic backgrounds entailing precocious lncRNA termination: *asp1-H397A*, *seb1-G476S*, *rad24*∆, *aps1*∆, *duf89*∆, and CTD mutants *S7A*, *S5-S5A*, and *P6-P6A*. Our findings that *snf22*∆ and *snf22-*(*D996A-E997A*) block *pho1* mRNA production in the *pho1*-derepressive *asp1-H397A*, *seb1-G476S*, and *rad24*∆ mutants without reversing the absence of the *PHO*-interfering *prt* lncRNA point to the *PHO* mRNA promoters as the sites of Snf22 action. The observations that *snf22-*(*D996A-E997A*) is not synthetically lethal or sick with *PHO* hyper-repressive 3′-processing/termination mutations support our inference that Snf22 is not acting at that step of the transcription cycle.

The present results highlighting Snf22 and Sol1, and by extension the SWI/SNF complex, in expression of the *PHO* mRNAs resonates with the foundational studies of fission yeast phosphate homeostasis by the Wykoff laboratory, in which they identified a null allele of the SWI/SNF subunit Snf5 in a screen of a *S. pombe* gene deletion collection for strains defective in inducing Pho1 acid phosphatase in response to phosphate starvation (47). They found that *snf5*∆ blocked the induction of *pho1* and *pho84* mRNAs during 4 h of phosphate starvation and prevented the constitutive derepression of Pho1 in phosphate-replete *aps1*∆ cells (47). Here we see that *snf22*∆ delays the induction of Pho1 expression during phosphate starvation and erases the Pho1 derepression by *aps1*∆. A microarray analysis of gene expression conducted by the Winston lab revealed nearly identical transcriptome profiles for the *snf22*∆ and *snf5*∆ mutants, in which *pho1* and *pho84* were both downregulated (28). Our RNA-seq analysis of *snf22*∆ and *snf22-*(*D996A-E997A*) revealed 65 other protein-coding genes that were downregulated in both strains.

We hypothesize that the ATP-dependent nucleosome remodeling activity of SWI/SNF is necessary to ensure full access of transcription factor Pho7 to its binding sites in the *PHO* mRNA promoters. In this regard, it is important to note that ChIP experiments had demonstrated the specific and strong physical association of Snf22 at the promoters of the *pho1*^+^ and *pex7*^+^ genes that stringently require Snf22 for their expression (28) (Fig. 7). The Allshire lab proposed that lncRNA transcription increases nucleosome density over the *pho1* and *tgp1* mRNA promoters and that nucleosome density is diminished during phosphate starvation, which allows access of Pho7 to the mRNA promoters (48). Our findings here that Snf22 is important to drive the *tgp1* and *pho1* promoters even when lncRNA synthesis is turned off suggests a slightly revised model whereby (i) the nucleosome distribution over the *PHO* mRNA promoters in the absence of SWI/SNF is not permissive for Pho7 binding; (ii) SWI/SNF rearranges the nucleosomes to allow Pho7 access, but lncRNA transcription across the mRNA promoter prompts ejection of Pho7 from the DNA; and (iii) when SWI/SNF is active, turning off the lncRNA promoter or precocious termination of lncRNA synthesis well upstream of the mRNA promoter relieves Pho7 ejection and allows Pho7 promoter occupancy to drive *PHO* mRNA transcription.

This mRNA promoter/Pho7-centric view of Snf22’s role in *PHO* gene regulation is fortified by our observations that (i) the fully derepressed *tgp1* promoter reporter, which contains a single Pho7 binding site, is more sensitive to loss of Snf22 than is the fully derepressed *pho1* promoter reporter that has two Pho7 binding sites in tandem and (ii) mutating one of the two Pho7 recognition sites in the *pho1* promoter makes mRNA synthesis more reliant on Snf22.

It is noteworthy that, in certain respects but not all parameters assayed, the ATPase mutant *snf22-*(*D996A-E997A*) exerted a stronger phenotype than did *snf22*∆. This was the case with respect to the completeness of suppression of the *asp1-STF6/9* growth defects, the degree of reversal of the *pho1* and *tgp1* depression phenotypes by *snf22-*(*D996A-E997A*) versus *snf22*∆, and in the *prt–pho1* plasmid reporter assays. One can speculate that the presence of an Snf22 ATPase-defective SWI/SNF complex at the *pho1* promoter might have a greater impact than the absence of SWI/SNF because it impedes another chromatin modeler (e.g., the essential INO80 or RSC complexes) from partially compensating for that absence. We note that Khavari et al. (49) had reported a dominant negative effect of an ATPase-defective Snf2 ortholog on transcription in *S. cerevisiae*.

## MATERIALS AND METHODS

### Spot tests of fission yeast growth

Cultures of *S. pombe* strains were grown in liquid yeast extract with supplements (YES) or enhanced pombe minimal glutamate with thiamine (ePMGT; prepared as described in Garg et al. [12]) medium until *A*_600_ reached 0.3–0.8. The cultures were adjusted to an *A*_600_ of 0.1 and aliquots (3 µL) of serial fivefold dilutions were spotted to YES or ePMGT agar. The plates were photographed after incubation for 2–2.5 days at 30°C, 34°C, and 37°C, 4 days at 25°C, and 6 days at 20°C.

### Acid phosphatase activity

Cells were grown at 30°C in YES or ePMGT medium. Aliquots of exponentially growing cultures were harvested, washed with water, and resuspended in water. To quantify acid phosphatase activity, reaction mixtures (200 µL) containing 100 mM sodium acetate (pH 4.2), 10 mM *p*-nitrophenylphosphate, and cells (ranging from 0.01 to 0.1 *A*_600_ units) were incubated for 5 min at 30°C. The reactions were quenched by the addition of 1 mL of 1 M sodium carbonate, the cells were removed by centrifugation, and the absorbance of the supernatant at 410 nm was measured. Acid phosphatase activity is expressed as the ratio of *A*_410_ (*p*-nitrophenol production) to *A*_600_ (cells). The data are averages (±SEM) of at least three assays using cells from three independent cultures.

### Whole-genome sequencing

After PicoGreen quantification and quality control by Agilent BioAnalyzer, 500 ng aliquots of genomic DNA were sheared using a LE220-plus Focused-ultrasonicator (Covaris catalog # 500569), and sequencing libraries were prepared using the KAPA Hyper Prep Kit (Kapa Biosystems KK8504) with modifications. DNA libraries were subjected to size selection by mixture with 0.5 vol of AMPure XP beads (Beckman Coulter catalog # A63882) after post-ligation cleanup. Libraries were not amplified by PCR and were pooled equivolume for sequencing. Samples were run on a NovaSeq 6000 in a 150 bp/150 bp paired-end run using the NovaSeq 6000 SBS v1 Kit and an S1 flow cell (Illumina). The average number of read pairs per sample was 10 million.

### Mapping suppressor mutations

The FASTA file for the *S. pombe* genome was accessed from Pombase. The whole-genome sequencing data from the parental *STF6* and *STF9* cells and the suppressor mutants were aligned to the genome using Bowtie2 (50). The resulting SAM files were converted to BAM files using Samtools (51). Variants were identified by BCFtools (52) using the criteria of adjusted mapping quality = 40, minimum base quality = 20, and disabled probabilistic realignment for the computation of base alignment quality (BAQ) for considering variations or insertion-deletion events. The multi-allelic caller protocol was used for variant calling in BCFtools. Variants were annotated using SnpEff, with its in-built genome version for *S. pombe* (53). Variants were further filtered by removing all variations with an average mapping quality ≤25 (Phred scale). All variants present in the parental strain were excluded as non-causal mutations.

### *snf22* gene deletion and allelic replacement

To generate *snf22*∆, and strains harboring marked *snf22-WT* and *snf22-D996A-E997A* alleles, we first constructed pKS-based plasmids carrying *snf22* integration cassettes using standard PCR and cloning methods. For deletion of *snf22*, the cassette consisted of the following elements: (i) a 735 bp segment of genomic DNA 5′ of the *snf22*^+^ start codon (position +1) of the 5043 bp *snf22* ORF; (ii) a *kanMX* marker gene conferring resistance to G418; and (iii) a 870 bp *snf22* DNA fragment downstream of position +3640 of the *snf22* ORF. The integration cassette for allelic replacement of *snf22*^+^ by marked *snf22-WT* and the *snf22-D996A-E997A* alleles consisted of the following elements: (i) a *snf22* DNA segment (+2116 to +5125, extending 82 bp downstream of the stop codon); (ii) a 258 bp segment of genomic DNA containing the *nmt1*^+^ poly(A) site; (iii) a *kanMX* marker; and (iv) 720 bp of *snf22* genomic DNA downstream of position +5125. The *D996A-E997A* mutations were introduced into the *snf22-WT* integration cassette by two-stage overlap PCR and cloning. The integration cassettes, which were sequenced to exclude the presence of unwanted mutations, were then excised from plasmids and transfected into diploid *S. pombe* cells. G418-resistant transformants were selected and analyzed by Southern blotting to verify correct integrations at the *snf22* locus. The presence of the D996A-E997A mutation was verified by PCR-amplifying and sequencing a segment of *snf22* DNA. Confirmed heterozygous diploids were sporulated and subjected to random spore analysis (54), whereby spores were plated on YES agar and incubated at 30°C. Upon replica plating to G418-containing medium, resistant haploid *snf22*∆, *snf22-WT*, and *snf22-D996A-E997A* progeny were recovered and comprised approximately half of the total progeny that grew on YES agar. Using the marker switching methodology (55), we also generated strains in which the *kanMX* module (marking *snf22* alleles) was replaced by the antibiotic resistance modules *hygMX* or *natMX*, or by *ura4MX* for selection on medium lacking uracil.

### *sol1* and *swr1* deletions

We generated *sol1*∆ and *swr1*∆ strains, in which the entire *sol1* ORF, or the coding sequence for amino acids 1–777 of the 1288-aa Swr1 protein, were replaced by *kanMX* and *natMX* marker genes, respectively. We first constructed integration cassettes in bacterial plasmids. In the *sol1*∆ gene disruption cassette, the *kanMX* resistance module is flanked by 521 bp and 584 bp DNA segments corresponding to genomic sequences upstream and downstream of the *sol1* ORF. The *swr1*∆ integration cassette consisted of a 715 bp segment of genomic DNA upstream of the *swr1*^+^ start codon, followed by the *natMX* module and a 530 bp DNA segment 3′ of nucleotide 2331 in the *swr1* ORF. The cassettes were excised from the plasmids and transfected into diploid *S. pombe* cells. Geneticin-resistant or nourseothricin-resistant transformants were selected and analyzed by Southern blotting to confirm correct integration at the *sol1* or *swr1* locus. Confirmed heterozygous diploids were sporulated and *sol1*∆*::kanMX* or *swr1*∆::*natMX* haploid progeny were selected.

### Construction of double mutants

Standard genetic methods were employed to generate haploid strains harboring mutations/deletions in two differently marked genes. In brief, pairs of haploids with null or missense mutations were mixed on malt agar to allow mating and sporulation, and the mixture was then subjected to random spore analysis (54). Spores (~1,500) were plated on YES agar and media selective for marked mutant alleles; the plates were incubated at 30°C for up to 5 days to allow slow-growing progeny to germinate and form colonies. At least 500 viable progeny were screened by replica-plating for the presence of the second marker gene, or by sequentially replica-plating from YES to selective media. Growth phenotypes of viable double mutant strains were assessed in parallel with the parental single-mutant and wild-type cells at different temperatures (20°C to 37°C) by spotting as described above.

### Reverse transcriptase quantitative PCR analysis

Total RNA was isolated from *S. pombe* wild-type, *STF6*, *snf22*∆, *snf22-*(*D996A-E997A*), *STF6 snf22*∆, and *STF6 snf22-*(*D996A-E997A*) cells grown in liquid ePMGT medium at 30°C to an *A*_600_ of 0.5 to 0.8 (three independent cultures for each strain). Cells were harvested by centrifugation and total RNA was extracted via the hot phenol method. The RNAs were treated with DNase I, extracted serially with phenol:chloroform and chloroform, and then precipitated with ethanol. The RNAs were resuspended in 10 mM Tris HCl (pH 6.8), 1 mM EDTA and adjusted to a concentration of 500 ng/µL. Reverse transcription was performed with 2 µg of this RNA template plus oligo(dT)_18_ and random hexamer primers by using the Maxima First Strand cDNA synthesis kit (Thermo Scientific). After cDNA synthesis for 30 min at 55°C, the reverse transcription reaction mixtures were diluted 10-fold with water. Aliquots (2 µL) were used as templates for gene-specific quantitative PCRs (qPCRs) directed by the sense and antisense primers listed in Table S6. The qPCRs were constituted with the Maxima SYBR Green/ROX master mix (Thermo Scientific) and monitored with an Applied Biosystems QuantStudio 6 Flex Real-Time PCR system. The qPCRs were performed in triplicate for each cDNA population. The level of individual cDNAs was calculated relative to that of act1 cDNA by the comparative Ct method (56). The actin-normalized levels of the *PHO* transcripts in wild-type cells were assigned a value of 1.0 and the mRNA levels in each mutant were then normalized to the wild-type control value.

### Transcriptome profiling by RNA-seq

RNA was isolated from *S. pombe* wild-type, *snf22*∆*,* and *snf22-*(*D996A-E997A*) cells that were grown in liquid YES medium at 30°C to an *A*_600_ of 0.5 to 0.6. Cells were harvested by centrifugation and total RNA was extracted via the hot phenol method. The integrity of total RNA was gauged with an Agilent Technologies 2100 Bioanalyzer. The Illumina TruSeq stranded mRNA sample preparation kit was used to purify poly(A)^+^ RNA from 500 ng of total RNA and to carry out the subsequent steps of poly(A)^+^ RNA fragmentation, strand-specific cDNA synthesis, indexing, and amplification. Indexed libraries were normalized using Qubit and Bioanalyzer and pooled in equimolar amounts for paired-end sequencing performed using a NovaSeq 6000 system. FASTQ files bearing paired-end reads of length 51 bases were mapped to the *S. pombe* genome (ASM294v2.28) using HISAT2-2.1.0 with default parameters (57). The resulting SAM files were converted to BAM files using Samtools (51). Count files for individual replicates were generated with HTSeq-0.10.0 (58) using exon annotations from Pombase (GFF annotations, genome-version ASM294v2; source “ensembl”). RPKM analysis and pairwise correlations (Tables S1 and S2) were performed as described previously (46). Differential gene expression and fold change analysis were performed in DESeq2 (59). Cut-off for further evaluation was set for genes that had an adjusted *P*-value (Benjamini-Hochberg corrected) of ≤0.05 and were up or down by at least twofold in *snf22∆* or *snf22-*(*D996A-E997A*) versus wild type. Genes were further filtered on the following criteria: (i) genes that were ≥2-fold up and the average normalized read count for the mutant strain was ≥100 and (ii) genes that were ≥2-fold down and the average normalized read count for the wild-type strain was ≥100.

### Northern blot analyses

Total RNA was extracted via the hot phenol method from 6 *A*_600_ units of yeast cells that had been grown exponentially in YES +G418 (for selection of *kanMX* plasmids) to *A*_600_ of 0.5 to 0.8 at 30°C. Aliquots (10 µg) of total RNA were resolved by electrophoresis through a 1.2% agarose/formaldehyde gel. After visualization of ethidium bromide-stained rRNAs under UV light, the gel contents were transferred to a Hybond-XL membrane (GE Healthcare). Hybridization was performed with a 5′ ^32^P-labeled ssDNA complementary to nucleotides 160–202 or 84–115 downstream of the *prt* or *pho1* transcription start sites, respectively, using a commercial hybridization buffer (Invitrogen ULTRAhyb-Oligo). Hybridized probes were visualized by autoradiography and quantified in ImageQuant after scanning the blot with a phosphorimager.

### Phosphate starvation response

Wild-type and *snf22*∆ cells were grown at 30°C in YES medium to *A*_600_ of 0.5 to 0.8. The cells were harvested, washed with water, and adjusted to *A*_600_ of ∼0.3 in ePMGT medium without phosphate (12) after withdrawing an aliquot to measure Pho1 activity (time 0). Acid phosphatase Pho1 activity was assayed every hour during a 7-h period of phosphate starvation.

### Fission yeast strains used in this study

See Table S7 for fission yeast strains.

## Data Availability

The RNA-seq data in this publication have been deposited in NCBI's Gene Expression Omnibus and are accessible through GEO Series accession number GSE245138.

## SUPPLEMENTAL MATERIAL

Supplemental tables and figures mbio.01252-24-s0001.pdf Tables S1, S2, S6, and S7 and Figures S1 to S4.

Table S3 mbio.01252-24-s0002.pdf Coding genes that are down- and upregulated in *snf22*∆.

Table S4 mbio.01252-24-s0003.pdf Coding genes dysregulated in *snf22* ATPase mutant.

Table S5 mbio.01252-24-s0004.pdf Overlap of dysregulated coding genes between *snf22*∆ and *snf22* ATPase mutants.

ASM does not own the copyrights to Supplemental Material that may be linked to, or accessed through, an article. The authors have granted ASM a non-exclusive, world-wide license to publish the Supplemental Material files. Please contact the corresponding author directly for reuse.
