# Supplementary material for: Suppression of inositol pyrophosphate toxicosis and hyper-repression of the fission yeast PHO regulon by loss-of-function mutations in chromatin remodelers Snf22 and Sol1
Source: mBio. 2024 Jun 20;15(7):e01252-24. doi: 10.1128/mbio.01252-24 (PMC11253589; doi:10.1128/mbio.01252-24)
Supplement: Supplemental tables and figures — Tables S1, S2, S6, and S7 and Figures S1 to S4. [file mbio.01252-24-s0001.pdf]

Supplemental Material

Suppression of inositol pyrophosphate toxicosis and hyper-repression of the fission yeast *PHO* regulon by loss-of-function mutations in chromatin remodeler Snf22

Beate Schwer, Aleksei Innokentev, Ana M. Sanchez, Angad Garg, and Stewart Shuman

Supplemental Tables S1, S2, S6, S7.

Supplemental Figures S1, S2, S3, S4.

Supplemental Table S3, S4, and S5 are separate pdf files

Sample	Total Paired Reads	Mapped Reads
WT (1)	27492840	25516105 (93%)
WT (2)	23412725	22508994 (96%)
WT (3)	19131272	18207232 (95%)
*snf22*Δ (1)	25160600	24476232 (97%)
*snf22*Δ (2)	25844234	24973283 (97%)
*snf22*Δ (3)	23014100	22114249 (96%)
snf22-(D996A-E997A) (1)	24221518	23087951 (95%)
snf22-(D996A-E997A) (2)	25524803	23990762 (94%)
snf22-(D996A-E997A) (3)	24052523	22671908 (94%)

Table S1. RNA-seq read counts for triplicate biological replicates.

Sample pairs	Pearson Coefficient
WT (1) vs (2)	0.980
WT (2) vs (3)	0.978
WT (1) vs (3)	0.980
*snf22*Δ (1) vs (2)	0.988
*snf22*Δ (2) vs (3)	0.985
*snf22*Δ (1) vs (3)	0.987
snf22-(D996A-E997A) (1)	0.986
snf22-(D996A-E997A) (2)	0.986
snf22-(D996A-E997A) (3)	0.984

Table S2. RNA-seq data reproducibility between biological replicates.

Gene	Strand	Primer Sequence
act1	sense	5'–AAGTACCCCATTGAGCACGG
	antisense	5'–CAGTCAACAAGCAAGGGTGC
tgp1	sense	5'–CCTGGTGCCTTACTTGGAGG
	antisense	5'–AGCAAACCAATGTTGCCACC
pho1	sense	5'–GCAGCGCGTTGTAGATTCTG
	antisense	5'–TCGAGAGCAGGGGTAGTGAA
pho84	sense	5'–ATTGCAACCGCATTCGGACT
	antisense	5'–GCACCCTCTTCGACTTGGTC

Table S6. Oligonucleotide primers used for qPCR analyses.

Table S7. List of *S. pombe* strains used in this study.

Strain	Genotype	Source
JS77	*h- leu1-32 ura4-D18 his3-D1 ade6-m216*	1
JS78	*h+ leu1-32 ura4-D18 his3-D1 ade6-m210*	1
BS204	*h- STF7 [asp1-H686Y] rpb1::kanMX*	2
AS2185	*h+ asp1-H397A::natMX*	3
AS1917	*h- aps1Δ::natMX*	3
AS2548	*h- duf89Δ::natMX*	4
BS272	*h- seb1-G476S::hygMX*	5
AGP141	*h+ rad24Δ::natMX*	6
BS255	*h+ SST-710 [asp1-H686Y] rpb1-WT::kanMX snf22-(+1fs aa 1563)*	this study
BS520	*h+ snf22Δ::kanMX*	this study
BS649	*h+ snf22Δ::natMX*	this study
BS650	*h+ snf22Δ::ura4MX*	this study
BS534	*h- snf22Δ::kanMX STF6::hygMX [asp1-W386*]*	this study
BS536	*h- snf22Δ::kanMX STF9::hygMX [asp1-W493*]*	this study
BS597	*h+ snf22Δ::kanMX asp1-H397A::hygMX*	this study
BS614	*h+ snf22Δ::kanMX aps1Δ::natMX*	this study
BS572	*h- snf22Δ::kanMX rpb1-WT::natR*	this study
BS575	*h- snf22Δ::kanMX rpb1-S5•S5A::natMX*	this study
BS576	*h- snf22Δ::kanMX rpb1-P6•P6A::natMX*	this study
BS584	*h- snf22Δ::kanMX rpb1-S7A::natMX*	this study
BS599	*h+ snf22Δ::kanMX seb1-G476S::hygMX*	this study
BS609	*h- snf22Δ::kanMX duf89Δ::natMX*	this study
BS600	*h- snf22Δ::kanMX rad24::natMX*	this study
BS631	*h- snf22-WT::kanMX*	this study
BS633	*h- snf22-D996A-E997A::kanMX*	this study
BS654	*h- snf22-D996A-E997A::hygMX*	this study
BS655	*h- snf22-D996A-E997A::natMX*	this study
BS635	*h+ snf22-WT::kanMX rpb1-S5•S5A::natMX*	this study
BS637	*h+ snf22-WT::kanMX asp1-H397A::natMX*	this study
BS638	*h- snf22-WT::kanMX erh1Δ::natMX*	this study
BS645	*h+ snf22-WT::kanMX seb1-G476S::hygMX*	this study
BS647	*h+ snf22-D996A-E997A::kanMX STF6::hygMX [asp1-W386*]*	this study
BS648	*h- snf22-D996A-E997A::kanMX STF9::hygMX [asp1-W493*]*	this study
BS639	*h+ snf22-D996A-E997A::kanMX rpb1-S5•S5A::natMX*	this study
BS641	*h- snf22-D996A-E997A::kanMX asp1-H397A::natMX*	this study
BS642	*h- snf22-D996A-E997A::kanMX erh1Δ::natMX*	this study
BS646	*h? snf22-D996A-E997A::kanMX seb1-G476S::hygMX*	this study
BS629	*h+ snf22Δ::natMX [prt2-pho84-prt-pho1]Δ::hygMX*	this study
BS687	*h+ snf22-D996A-E997A::natMX [prt2-pho84-prt-pho1]Δ::ura4MX*	this study

BS657	*h- seb1-G476S::hygMX ∆snf22::natMX [prt2-pho84-prt-pho1]Δ::ura4MX*	this study
BS658	*h+ asp1-H397A::natR snf22∆::ura4MX [prt2-pho84-prt-pho1]Δ::hygMX*	this study
BS659	*h+ snf22∆::ura4MX rad24∆::natMX [prt2-pho84-prt-pho1]Δ::hygMX*	this study
BS688	*h- snf22-D996A-E997A::natMX seb1-G476S::hygMX [prt2-pho84-prt-pho1]Δ::ura4MX*	this study
BS689	*h+ snf22-D996A-E997A::hygMX rad24::natMX [prt2-pho84-prt-pho1]Δ::ura4MX*	this study
BS690	*h+ snf22-D996A-E997A::hygMX asp1-H397A::natMX [prt2-pho84-prt-pho1]Δ::ura4MX*	this study
BS647	*h+ snf22-D996A-E997A::kanMX STF6::hygMX [asp1-W386*]*	this study
BS648	*h+ snf22-D996A-E997A::kanMX STF9::hygMX [asp1-W493*]*	this study
BS662	*h+ snf22-WT::kanMX ppn1∆::natMX*	this study
BS663	*h+ snf22-WT::kanMX swd22∆::natMX*	this study
BS664	*h- snf22-WT::kanMX asp1∆::natMX*	this study
BS665	*h- snf22-WT::kanMX asp1-D333A::natMX*	this study
BS666	*h- snf22-WT::kanMX ssu72-C13S::hygMX*	this study
BS667	*h+ snf22-WT::kanMX rhn1∆::hygMX*	this study
BS668	*h+ snf22-WT::kanMX spx1∆::ura4MX*	this study
BS669	*h+ snf22-D996A-E997A::kanMX rpb1-T4A::natMX*	this study
BS671	*h+ snf22-D996A-E997A::kanMX ppn1∆::natMX*	this study
BS672	*h- snf22-D996A-E997A::kanMX swd22∆::natMX*	this study
BS673	*h- snf22-D996A-E997A::kanMX asp1∆::natMX*	this study
BS674	*h- snf22-D996A-E997A::kanMX asp1-D333A::natMX*	this study
BS675	*h- snf22-D996A-E997A::kanMX ssu72-C13S::hygMX*	this study
BS676	*h+ snf22-D996A-E997A::kanMX rhn1∆::hygMX*	this study
BS681	*h+ snf22-D996A-E997A::kanMX spx1∆::ura4MX*	this study
BS660	*h- snf22-WT::kanMX rpb1-T4A::natMX*	this study
BS1211	*h+ swr1∆::natMX*	this study
BS1212	*h- swr1∆::natMX STF6::hygMX [asp1-W386*]*	this study
BS1213	*h+ swr1∆::natMX STF9::hygMX [asp1-W493*]*	this study
BS1214	*h? swr1∆::natMX snf22∆::kanMX*	this study
BS1234	*h- swr1∆::natMX snf22-D996A-E997A::kanMX*	this study
IAP99	*h- sol1Δ::kanMX*	this study
IAP103	*h- sol1Δ::kanMX STF6::hygMX [asp1-W386*]*	this study
IAP104	*h- sol1Δ::kanMX STF9::hygMX [asp1-W493*]*	this study

All strains are derived from the parental strains (JS77 and JS78). The strains are *leu1-32 ura4-D18 his3-D1* and either *ade6-m216 or ade6-m210*. The *asp1* alleles in the *STF* mutants are specified in brackets. Mating types were determined by mixing cells with each of the parental *h+* and *h-* strains on malt agar and observing tetrads after 24-48 h of incubation. *h?* indicates that no tetrads were observed.

1. Pei Y, Du H, Singer J, St Amour C, Granitto S, Shuman S, Fisher RP. (2006) Cyclin-dependent kinase 9 (Cdk9) of fission yeast is activated by the CDK-activating kinase Csk1, overlaps functionally with the TFIIH-associated kinase Mcs6, and associates with the mRNA cap methyltransferase Pcm1 in vivo. *Mol Cell Biol* 26:777-788.

2. Garg A, Shuman S, Schwer B. (2020) A genetic screen for suppressors of hyper-repression of the fission yeast *PHO* regulon by Pol2 CTD mutation T4A implicates inositol 1-pyrophosphates as agonists of precocious lncRNA transcription termination. *Nucleic Acids Res* 48:10739-10752.
3. Sanchez AM, Garg A, Shuman S, Schwer B. (2019) Inositol pyrophosphates impact phosphate homeostasis via modulation of RNA 3' processing and transcription termination. *Nucleic Acids Res* 47: 8452-8469.
4. Sanchez AM, Garg A, Schwer B, Shuman S. (2023) Duf89 abets lncRNA control of fission yeast phosphate homeostasis via its antagonism of precocious lncRNA transcription termination. *RNA* 29: 808-825.
5. Schwer B, Garg A, Jacewicz A, Shuman S. (2021) Genetic screen for suppression of transcriptional interference identifies a gain-of-function mutation in Pol2 termination factor Seb1. *Proc Natl Acad Sci USA* 118: e2108105118.
6. Garg A, Shuman S, Schwer B. (2022) Genetic screen for suppression of transcriptional interference reveals fission yeast 14-3-3 protein Rad24 as an antagonist of precocious Pol2 transcription termination. *Nucleic Acids Res* 50: 803-819

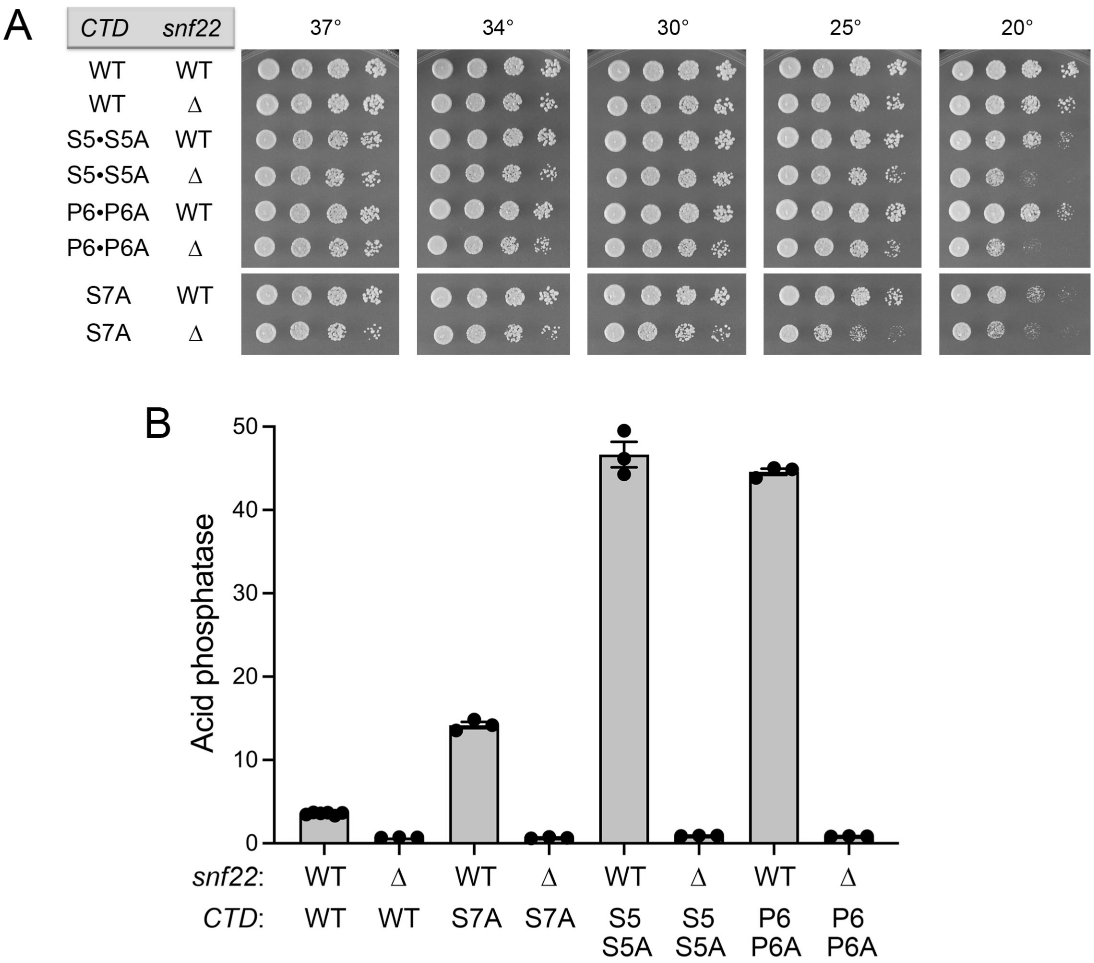

Figure S1. <u>*snf22*Δ interdicts Pho1 derepression by Pol2 CTD mutations</u>. (A) Serial 5-fold dilutions of fission yeast strains (as specified on the left) were spot tested for growth on YES agar at the indicated temperatures. (B) The indicated strains were assayed for Pho1 acid phosphatase activity.

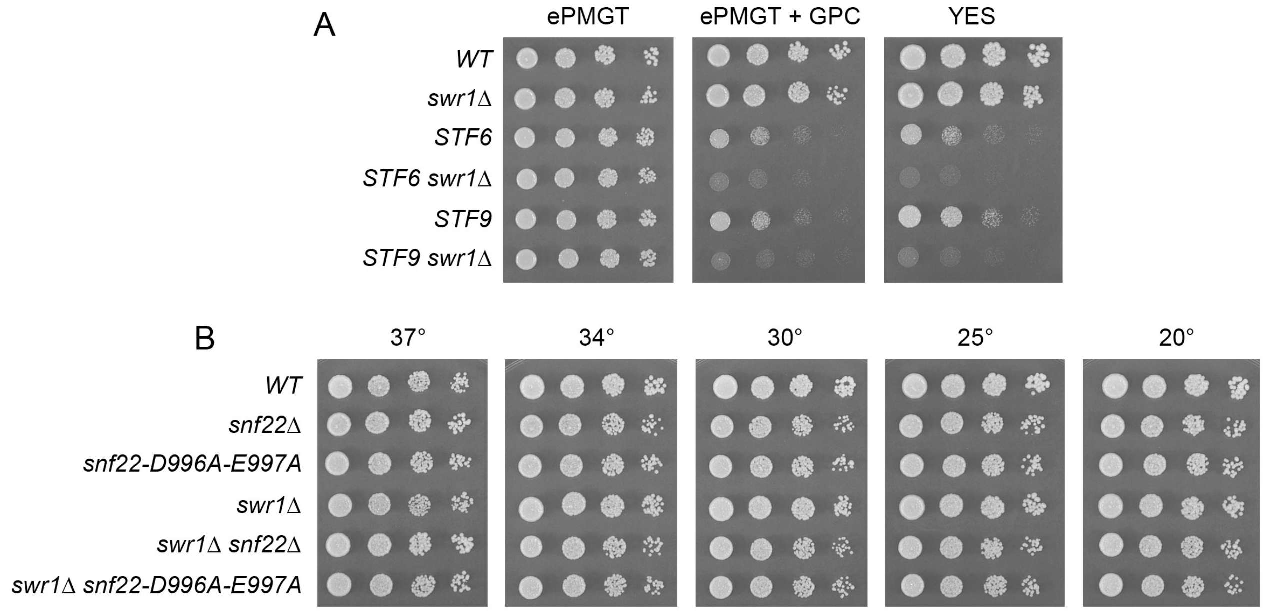

Figure S2. Deletion of chromatin remodeling ATPase Swr1 does not suppress *asp1-STF* toxicosis. (A) Serial 5-fold dilutions of fission yeast strains (as specified on the left) were spot-tested for growth on ePMGT agar, ePMGT agar plus 250 μM GPC, and YES agar at 30˚C. (B) Serial 5-fold dilutions of fission yeast strains with the indicated *snf22* and *swr1* alleles were spot-tested for growth on YES agar at the indicated temperatures.

gene	log2 change		function
	snf22-(D996A-E997A)	*snf22Δ*	
str3 *	6.45		heme transporter
ght1 *	4.69	5.10	glucose transporter
mug14	3.97	5.85	adducin family
SPCC1235.18	3.63	4.11	
SPBPB21E7.11	3.51		pombe specific protein
alr2 *	3.43	3.92	alanine racemase
ght5 *	3.43	4.00	hexose transporter
ght4 *	3.05	5.22	hexose transporter
SPCC1235.17	3.02	4.40	
mfm1	2.84		M-factor precursor
cmt2	2.83	1.37	O-methyltransferase
ght7	2.71		hexose transporter
mam2	2.67		pheromone receptor
ght3 *	2.24	4.76	gluconate transporter
frp1 *	2.20		ferric-chelate reductase
fio1 *	2.18	1.82	iron transport oxidase
str1 *	2.02		siderophore-iron transporter
SPCC1739.08c *	1.98	5.69	short chain dehydrogenase
gdt1		4.75	Golgi calcium and manganese antiporter
rsv1 *		4.69	DNA-binding transcription repressor
gcd1		4.32	glucose dehydrogenase
gal10	1.51	2.95	UDP-glucose 4-epimerase
rbd3	1.36	2.50	rhomboid family protease
plb1 *	1.97	2.44	phospholipase B
SPAC11D3.19		2.33	pombe specific protein
mug147	1.79	2.19	pombe specific protein
SPAC4H3.03c *	1.36	2.17	glucan 1,4-alpha-glucosidase
SPAC27D7.11c *		2.16	But2 family protein
SPAC17A2.11	1.47	2.15	
gal7	1.66	2.05	galactose-1-phosphate uridylyltransferase

Figure S3. List of the coding genes that were upregulated by at least 4-fold (log2 change of ≥2.0) in *snf22-(D996A-E997A)* cells (n=17) or *snf22*Δ cells (n=21), of which 18 genes were coordinately up by at least 2-fold (log2 change of ≥1.0) in both strains. Fourteen of the most strongly upregulated transcripts observed by RNA-seq had been detected by microarray analysis of *snf22*Δ are denoted by asterisks.

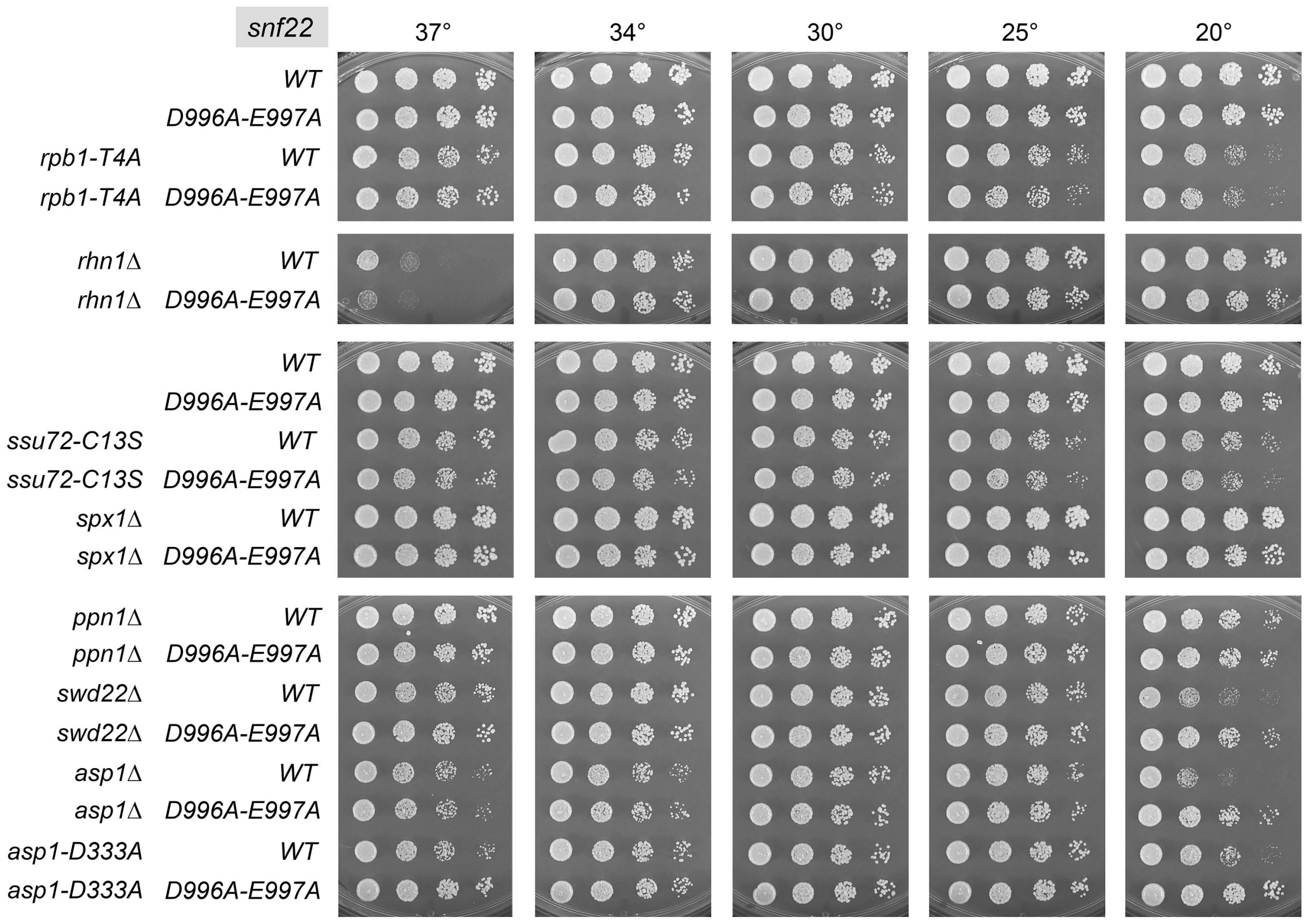

Figure S4. Lack of synthetic growth defects in *snf22-(D996A-R997A) CPF/rhn1/spx1/asp1* double mutants. Serial 5-fold dilutions of fission yeast strains (as specified on the left) were spot-tested for growth on YES agar at the indicated temperatures.
