# Supplementary material for: Suppression of inositol pyrophosphate toxicosis and hyper-repression of the fission yeast PHO regulon by loss-of-function mutations in chromatin remodelers Snf22 and Sol1
Source: mBio. 2024 Jun 20;15(7):e01252-24. doi: 10.1128/mbio.01252-24 (PMC11253589; doi:10.1128/mbio.01252-24)
Supplement: Table S3 — Coding genes that are down- and upregulated in snf22∆. [file mbio.01252-24-s0002.pdf]

Table S3 legend.

Lists of the protein coding genes that were downregulated by at least 2-fold (log2 change of –1.0 or greater) in *snf22*Δ cells and the protein-coding genes that were upregulated by at least 2-fold (log2 change of 1.0 or greater) in *snf22*Δ cells. Genes that were downregulated or upregulated by at least 4-fold (log2 changes of at least –2.0 or 2.0, respectively) are shaded.

Systematic ID	Gene name	Product description	log2FoldChange	pvalue
SPBPB2B2.01		amino acid transmembrane transporter	-7.194690502	7.259E-139
SPBPB2B2.06c		extracellular 5'-nucleotidase, human NT5E family	-7.074623586	2.0053E-50
SPAC17D4.01	pex7	peroxin-7	-5.938387246	0
SPBP4G3.02	pho1	extracellular acid phosphatase Pho1	-5.796493386	0
SPAC1039.02		extracellular 5'-nucleotidase, human NT5E family	-4.756527895	1.2381E-76
SPBPB21E7.07	aes1	phenazine biosynthesis PhzF protein family	-4.644251876	8.8148E-79
SPBC8E4.01c	pho84	plasma membrane inorganic phosphate transmembrane transporter	-4.140356559	0
SPAC2E1P3.05c		fungal cellulose binding domain protein	-3.59764038	3.765E-188
SPBPB2B2.05		class I glutamine amidotransferase family protein	-3.26800923	8.3042E-28
SPBC29B5.02c	isp4	plasma membrane OPT oligopeptide transmembrane transporter family Isp4	-3.093839506	4.5445E-65
SPAPB24D3.07c		Schizosaccharomyces pombe specific protein	-2.865718039	1.6497E-81
SPBPB21E7.08		pseudogene	-2.72375098	1.19E-115
SPBPB8B6.04c	grt1	DNA-binding transcription factor Grt1	-2.708344011	1.947E-37
SPAC3C7.14c	obr1	NAD(P)H dehydrogenase (quinone)	-2.702607195	1.463E-149
SPBC26H8.11c	the4	acyl-coenzyme A thioesterase The4	-2.638488508	1.3367E-85
SPBC23G7.13c		plasma membrane urea transmembrane transporter	-2.605692425	3.5526E-16
SPBC1861.02	abp2	unknown protein, may bind replication origins Abp2	-2.445120993	2.122E-102
SPAC513.07		flavonol reductase/cinnamoyl-CoA reductase family	-2.426811196	5.369E-146
SPBC1271.08c		Schizosaccharomyces pombe specific protein	-2.404183554	2.6296E-51
SPBPB10D8.01		cysteine transmembrane transporter	-2.34632991	3.3566E-75
SPCPB1C11.03		cysteine transmembrane transporter	-2.311872298	1.814E-100
SPCC70.08c		methyltransferase	-2.303954339	6.4934E-15
SPAC110.01	ppk1	serine/threonine protein kinase Ppk1	-2.178712157	2.1463E-86
SPBC36.03c	mfs3	plasma membrane spermidine transmembrane transporter Mfs3	-2.17183271	2.5345E-95
SPAC5H10.03		phosphoglycerate mutase/6-phosphofructo-2-kinase family	-2.096779793	1.6248E-63
SPCC584.16c		Schizosaccharomyces specific protein	-2.051905468	2.8696E-76
SPCC1223.03c	gut2	glycerol-3-phosphate dehydrogenase Gut2	-2.018482423	9.0118E-33
SPCC18B5.01c	bfr1	plasma membrane brefeldin A efflux transporter Bfr1	-2.015237441	1.0467E-17
SPAC1002.16c		carboxylic acid transmembrane transporter	-1.974612731	4.0429E-34
SPAC513.03	mfm2	M-factor precursor Mfm2	-1.951286122	1.1969E-31
SPCC569.05c		plasma membrane spermidine family transmembrane transporter	-1.857010778	3.4303E-25
SPBC8E4.12c	ecl3	extender of chronological lifespan protein Ecl3	-1.855322143	1.8423E-31
SPBC1347.11	sro1	stress responsive orphan 1	-1.838401819	4.7044E-09
SPBC1861.01c	cnp3	CENP-C ortholog Cnp3	-1.816415995	1.4022E-33
SPAC29B12.10c	pgt1	plasma membrane glutathione transmembrane transporter Pgt1	-1.754059162	1.3836E-49
SPBC1289.14		adducin	-1.743281859	3.5134E-11
SPAC27D7.03c	mei2	RNA-binding protein involved in meiosis Mei2	-1.69579543	5.5272E-64
SPAC5H10.06c	adh4	alcohol dehydrogenase Adh4	-1.692115871	2.4288E-44
SPCC757.07c	ctt1	catalase	-1.679160684	8.4931E-23
SPCC965.14c		cytosine deaminase	-1.653490604	2.2541E-22
SPAC8E11.10		sorbose reductase	-1.63639658	2.9768E-72
SPAC1F8.06	pfl8	cell surface glycoprotein, flocculin Pfl8	-1.629479707	5.5024E-48
SPAC7D4.08		Schizosaccharomyces pombe specific protein	-1.598536712	3.0184E-15
SPBP8B7.05c	nce103	carbonic anhydrase	-1.590294667	8.1699E-45
SPBC1271.07c		N-acetyltransferase	-1.580140845	2.381E-35
SPAC1039.01		amino acid transmembrane transporter	-1.555512958	7.7052E-45
SPBC713.11c	pmp3	plasma membrane proteolipid Pmp3	-1.534704716	2.898E-24
SPBC428.02c	eca39	branched chain amino acid aminotransferase Eca39	-1.533146195	1.0556E-33
SPBC1685.17		Schizosaccharomyces pombe specific protein	-1.518920313	1.189E-19
SPBC1685.13	fhn1	eisosome assembly protein Fhn1	-1.501662425	3.9899E-06
SPBC36.01c		spermidine family transmembrane transporter	-1.498346378	3.3755E-57
SPAC21E11.04	aca1	L-azetidine-2-carboxylic acid acetyltransferase Aca1	-1.489329396	1.0623E-19
SPCC794.12c	mae2	malic enzyme, malate dehydrogenase (oxaloacetate decarboxylating), Mae2	-1.485456465	6.3502E-52
SPBC11C11.05		conserved fungal cell wall protein, Kre9/Knh1 family	-1.478647677	3.8112E-09
SPBC24C6.09c		phosphoketolase family protein	-1.459861738	2.1522E-08
SPAC17C9.16c	mfs1	plasma membrane spermidine transmembrane transporter Mfs1	-1.450881815	7.4879E-67
SPAC26H5.09c		oxidoreductase involved in NADPH regeneration	-1.443767592	2.7962E-45
SPBPJ4664.02		crazy cell surface glycoprotein	-1.434120161	1.2719E-25
SPBC25B2.08		Schizosaccharomyces pombe specific protein	-1.423193845	7.1428E-29
SPAC922.04		Schizosaccharomyces specific protein	-1.422096947	1.2082E-28
SPCC70.12c	ecl1	extender of chronological lifespan protein Ecl1	-1.410643163	2.7926E-07
SPAC1002.17c	urg2	uracil phosphoribosyltransferase	-1.393594584	4.1417E-06
SPAC1687.16c	erg31	C-5 sterol desaturase Erg31	-1.39056278	6.1925E-44
SPAC3H1.11	hsr1	DNA-binding transcription factor Hsr1	-1.384936436	6.0811E-53
SPAC31G5.11	pac2	cAMP-independent regulatory protein Pac2	-1.37452297	5.8386E-45
SPAC821.09	eng1	cell septum surface endo-1,3-beta-glucanase Eng1	-1.356482208	2.5767E-24
SPBC12C2.04		NAD binding dehydrogenase family protein	-1.354121813	9.8094E-20
SPAC9.10	thi9	plasma membrane thiamine/proton high affinity transmembrane transporter Thi9	-1.342345947	3.5386E-26
SPBC336.08	spc24	NMS complex subunit Spc24	-1.341121357	0.00129116
SPAC23H3.13c	gpa2	heterotrimeric G protein alpha-2 subunit Gpa2	-1.337601116	7.7135E-30
SPAC1B3.16c	vht1	plasma membrane vitamin H transmembrane transporter Vht1	-1.321050538	5.045E-104
SPAC10F6.16	igo1	serine/threonine protein phosphatase inhibitor, endosulfine Igo1	-1.303569284	1.0647E-25
SPBC32C12.02	ste11	DNA-binding transcription factor Ste11	-1.30289644	3.0392E-28
SPAPB24D3.09c	pdr1	ABC transmembrane transporter Pdr1	-1.289567863	5.3417E-07
SPAC31G5.02	rot1	ER chaperone Rot1	-1.282067645	1.51E-18
SPAC19G12.05	mce1	mitochondrial carrier, citrate	-1.278141251	1.1495E-31
SPBC428.05c	arg12	argininosuccinate synthase Arg12	-1.269622456	4.7332E-29
SPBC1711.15c		Schizosaccharomyces pombe specific protein	-1.26603827	2.8368E-12
SPAC11D3.17		DNA-binding transcription factor, zf-fungal binuclear cluster type	-1.243736275	2.0582E-14
SPAC3G6.05		mitochondrial Mpv17/PMP22 family protein 1	-1.243074836	5.1381E-24
SPBC21C3.19	rtc3	SBDS family protein Rtc3	-1.219176765	0.00052929
SPAC1093.01	ppr5	mitochondrial PPR repeat protein Ppr5	-1.214927692	9.4528E-25
SPBC1198.02	dea2	adenine deaminase Dea2	-1.202816304	6.8783E-40
SPCC1223.13	cbf12	DNA-binding transcription factor, CBF1/Su(H)/LAG-1 family Cbf12	-1.193057654	5.1569E-27
SPAC19E9.03	pas1	cyclin Pas1	-1.181134165	1.261E-14
SPBC1271.10c		transmembrane transporter	-1.177286501	1.5821E-12
SPAC57A7.05		transmembrane transporter	-1.169034947	3.4017E-05
SPAP7G5.06	per1	plasma membrane amino acid transmembrane transporter Per1	-1.145806249	1.7144E-14
SPCC965.13		plasma membrane pyridoxal family transmembrane transporter	-1.128798935	4.8944E-55
SPBC1683.01	pho841	plasma membrane inorganic phosphate transmembrane transporter	-1.122874638	5.7434E-37
SPBPB7E8.01		Schizosaccharomyces specific protein, predicted GPI anchor	-1.119196537	1.6125E-25
SPAC1002.18	urg3	DUF1688 family fungal conserved protein, implicated in uracil or riboflavin metabolism	-1.105767395	6.2148E-12
SPBC215.08c	arg4	arginine specific carbamoyl-phosphate synthase Arg4	-1.104601932	3.9641E-21
SPBC651.04		Schizosaccharomyces specific protein	-1.103046056	4.6751E-24
SPAC6F6.11c		pyridoxine-pyridoxal-pyridoxamine kinase	-1.097560366	1.8861E-30
SPBC56F2.09c	arg5	arginine specific carbamoyl-phosphate synthase subunit Arg5	-1.097350462	3.5085E-14
SPBC16E9.16c	lsd90	Lsd90 protein	-1.095140656	0.0010679
SPBC19C7.04c		DUF2406 family conserved fungal protein	-1.09455559	8.2398E-05
SPAC22A12.06c	fsh2	serine hydrolase-like, human TSTD2 and OVCA2 ortholog	-1.083136522	4.3289E-66
SPBPB21E7.09		L-asparaginase	-1.073240029	1.09E-25
SPAC521.03		short chain dehydrogenase, human DHRS7 family	-1.066403032	5.224E-14
SPCC1827.06c		aspartate semialdehyde dehydrogenase	-1.058037837	3.5848E-15
SPAC186.01	pfl9	cell surface glycoprotein, flocculin Pfl9, DIPSY family	-1.05550235	8.4801E-09
SPBC359.04c	pfl7	cell surface glycoprotein, flocculin Pfl7, DIPSY family	-1.052116794	1.3547E-10
SPAC23A1.03	apt1	adenine phosphoribosyltransferase (APRT) Apt1	-1.050602841	9.4949E-22
SPAC31G5.09c	spk1	MAP kinase Spk1	-1.047260817	1.2745E-16
SPCC1672.03c	gud1	guanine deaminase Gud1	-1.043677612	4.3767E-35
SPCC550.05	nse1	Smc5-6 complex ubiquitin-protein ligase E3 subunit Nse1	-1.040118196	6.8127E-06
SPBC16D10.06	zrt1	plasma membrane ZIP zinc transmembrane transporter Zrt1	-1.035410316	1.0216E-29
SPAC1F3.09	mug161	CwfJ family protein, splicing factor	-1.031651726	1.156E-33
SPAC222.08c	sno1	glutamine aminotransferase subunit Sno1	-1.031637883	1.4767E-16
SPAPB1E7.07	glt1	glutamate synthase Glt1	-1.022042204	6.8819E-22
SPCC11E10.01	cbl1	cystathionine beta-lyase Cbl1	-1.017528406	1.2681E-29
SPAC26F1.10c	pyp1	protein tyrosine phosphatase Pyp1	-1.015573832	5.4813E-05
SPBC365.16		mitochondrial membrane protein, conserved in yeast and apicomplexa	-1.007625473	9.5724E-40
SPBC887.17		plasma membrane guanine and adenine transmembrane transporter	-1.005466935	1.2907E-40
SPAC1399.04c	uck2	uracil phosphoribosyltransferase Uck2	-1.002445874	1.8547E-08

Systematic ID	Gene name	Product description	log2FoldChange	pvalue
SPBC359.06	mug14	adducin, involved in actin cytoskeleton organization	5.845140283	3.4696E-77
SPCC1739.08c		short chain dehydrogenase	5.688935216	2.072E-153
SPBC1683.08	ght4	plasma membrane hexose:proton symporter, unknown specificity Ght4	5.217563544	0
SPCC548.07c	ght1	plasma membrane high-affinity glucose:proton symporter Ght1	5.098431656	0
SPBC359.02	alr2	alanine racemase Alr2	4.919821835	1.295E-191
SPAC1F8.01	ght3	plasma membrane gluconate:proton symporter Ght3	4.758052367	4.4601E-29
SPAC186.05c	gdt1	Golgi calcium and manganese antiporter Gdt1	4.751684277	3.311E-159
SPBP4H10.09	rsv1	DNA-binding transcription repressor Rsv1	4.693362336	2.8256E-56
SPCC1235.17		dubious	4.399533214	7.096E-226
SPCC794.01c	gcd1	glucose dehydrogenase Gcd1	4.317992634	4.359E-186
SPCC1235.18		dubious	4.112958153	4.988E-153
SPCC1235.14	ght5	plasma membrane high-affinity glucose/fructose:proton symporter Ght5	3.996080451	4.523E-182
SPBPB2B2.12c	gal10	UDP-glucose 4-epimerase/aldose 1-epimerase Gal10	2.953863355	4.6534E-70
SPBP4H10.10	rbd3	mitochondrial rhomboid family protease	2.495921243	1.0094E-58
SPAC1A6.04c	plb1	phospholipase B homolog Plb1	2.44163559	8.244E-90
SPAC11D3.19		Schizosaccharomyces pombe specific protein	2.334045904	1.7184E-57
SPBC56F2.06	mug147	Schizosaccharomyces specific protein Mug147	2.189273124	1.6895E-18
SPAC4H3.03c		glucan 1,4-alpha-glucosidase	2.174071608	2.5757E-25
SPAC27D7.11c		But2 family protein, similar to cell surface molecules	2.156732628	5.0542E-13
SPAC17A2.11		dubious	2.149264058	2.5512E-24
SPBPB2B2.10c	gal7	galactose-1-phosphate uridylyltransferase Gal7	2.054184062	5.8485E-46
SPBPB2B2.13	gal1	galactokinase Gal1	1.97773818	1.9979E-52
SPBC32H8.02c	nep2	NEDD8 protease Nep2	1.922660976	6.6103E-70
SPBCPT2R1.08c	tlh2	RecQ type DNA helicase Tlh1	1.864231693	2.8688E-05
SPCC1840.12	opt3	OPT oligopeptide transmembrane transporter family protein Opt3	1.863590083	1.0733E-14
SPAC1F7.08	fio1	plasma membrane iron transport multicopper oxidase Fio1	1.823577771	5.9097E-27
SPAC29A4.12c	mug108	Schizosaccharomyces specific protein Mug108	1.822838232	1.0135E-07
SPCC4F11.05		dubious	1.728322498	7.8656E-15
SPCC737.04		UPF0300 family protein 6	1.691634488	1.6097E-10
SPBC215.10	odr1	HAD superfamily hydrolase, unknown role	1.671134265	5.5494E-81
SPCC1235.01		Schizosaccharomyces specific protein	1.660897923	2.219E-31
SPBPB7E8.02		PSP1 family protein	1.613058545	5.0914E-44
SPCC794.02	wtf5	wtf antidote-like meiotic drive suppressor Wtf5	1.592667017	6.877E-32
SPCC576.16c	wtf22	wtf element Wtf22 (frameshifted)	1.550488495	8.4524E-10
SPAC1039.09	isp5	amino acid transmembrane transporter Isp5	1.550233872	9.2314E-40
SPBC1289.16c	cao2	copper amine oxidase-like protein Cao2	1.546601392	1.8064E-16
SPBC1D7.02c	scr1	DNA-binding transcription repressor Scr1	1.500508629	5.0163E-88
SPAC1751.01c	gti1	gluconate transmembrane transporter inducer Gti1	1.482640993	3.4737E-49
SPAPB1A10.14	pof15	F-box protein	1.448421837	3.4012E-63
SPAC1A6.06c	meu31	Schizosaccharomyces specific protein Meu31	1.410605518	4.8655E-13
SPCC330.04c	mug135	DUF1773 family protein, with repeat expansion	1.408782069	7.6581E-08
SPBC660.16	gnd1	phosphogluconate dehydrogenase, decarboxylating	1.406291301	2.6134E-45
SPBC1198.14c	fbp1	fructose-1,6-bisphosphatase Fbp1	1.394427695	9.7583E-12
SPCC1906.04	wtf20	wtf antidote-like meiotic drive suppressor Wtf20	1.391602597	4.6137E-08
SPBPB21E7.04c	cmt2	O-methyltransferase, human COMT catechol homolog 2	1.37351221	0.00171955
SPAPB15E9.02c		dubious	1.363871364	7.3609E-08
SPAC20G4.03c	hri1	eIF2 alpha kinase Hri1	1.361998082	1.4157E-34
SPAPB15E9.06		dubious	1.352561285	4.3254E-18
SPBC23G7.10c		NADH-dependent flavin oxidoreductase	1.334726787	2.4343E-16
SPAC56F8.14c	mug115	Schizosaccharomyces pombe specific protein Mug115	1.311844458	3.5719E-31
SPCC794.03		amino acid transmembrane transporter	1.304561729	2.2683E-18
SPAPB24D3.04c	mag1	DNA-3-methyladenine glycosylase Mag1	1.301357499	1.7819E-14
SPBC19C2.06c	mug124	Schizosaccharomyces pombe specific protein	1.280945195	6.6366E-25
SPAC11D3.09		agmatinase	1.280397855	5.156E-15
SPAC17A2.10c		dubious	1.269108956	1.2591E-11
SPAC664.13		Schizosaccharomyces pombe specific protein	1.242273488	1.2046E-15
SPBC660.06	wwm2	WW domain containing conserved fungal protein Wwm2	1.236228657	8.0674E-23
SPBP26C9.02c	car1	arginase Car1	1.22635914	6.877E-29
SPBC19F8.06c	meu22	amino acid transmembrane transporter Meu22	1.215362286	2.9763E-13
SPAC23D3.17		protease inhibitor 178 family	1.214623801	5.2642E-09
SPAC13F5.03c	gld1	mitochondrial glycerol dehydrogenase Gld1	1.17638241	3.4255E-20
SPAC1F8.08		Schizosaccharomyces pombe specific protein	1.166636035	1.3878E-36
SPCC10H11.02	cwf23	DNAJ domain protein Cwf23	1.163241578	8.2034E-09
SPBC13E7.02	cwf24	ubiquitin-protein ligase E3/GCN5-related N acetyltransferase fusion protei	1.159251061	4.6744E-14
SPAC1399.06		dubious	1.150769498	8.8312E-33
SPAC3G9.11c	pdc201	pyruvate decarboxylase	1.119990762	5.3717E-14
SPAC27F1.03c	uch1	ubiquitin C-terminal hydrolase Uch1	1.105984803	2.7913E-12
SPCC320.14	sry1	serine racemase Sry1	1.101744138	3.0373E-27
SPAC3A12.02		mitochondrial inorganic diphosphatase	1.098679608	4.2512E-09
SPAC2E1P3.02c	amt3	plasma membrane ammonium transmembrane transporter Amt3	1.095155948	1.0686E-05
SPCC191.01		Schizosaccharomyces specific protein	1.092041664	3.9926E-15
SPBC32F12.15	tfb5	transcription factor TFIIH complex subunit Tfb5	1.08664044	3.7629E-09
SPBC713.09		Schizosaccharomyces specific protein	1.083294543	1.3486E-07
SPBC428.07	meu6	pleckstrin homology domain protein Meu6	1.07683007	9.9954E-07
SPAPB2B4.06		acyl-coenzyme A thioesterase	1.072453977	4.7142E-12
SPAC6C3.08	nas6	proteasome assembly chaperone, gankyrin	1.06350406	8.1734E-14
SPBC1348.12		DNA-binding transcription factor	1.0568035	3.7155E-05
SPBC1289.02c	uap2	U2 snRNP-associated protein Uap2	1.055221097	1.0748E-12
SPAC6B12.08	epr1	Atg8-interacting ER-phagy receptor Epr1	1.053636349	1.1111E-11
SPAC27D7.08c	mtl16	23S rRNA/U6 snRNA (adenine-N(6))-methyltransferase Mtl16	1.050295941	1.8223E-09
SPAC227.14	yfh7	uridine kinase Yfh7	1.047951579	5.8748E-29
SPAPB1A10.13		Schizosaccharomyces specific protein	1.047362222	8.7365E-19
SPAC1002.12c		succinate-semialdehyde dehydrogenase [NAD(P)+]	1.047252938	9.3617E-13
SPAC3H1.07	aru1	arginase Aru1	1.046869299	1.6148E-10
SPBC1A4.06c	tam41	mitochondrial phosphatidate cytidylyltransferase Tam41	1.034104164	3.4123E-11
SPAC9.13c	cwf16	splicing factor Cwf16	1.034064261	1.0668E-06
SPAC4A8.09c	cwf21	complexed with Cdc5 protein Cwf21	1.024268393	1.0684E-06
SPBC1778.02	rap1	shelterin complex telomere binding subunit Rap1	1.023018621	2.3978E-07
SPCC1739.09c	cox13	cytochrome c oxidase subunit VIa	1.018810234	1.56E-14
SPBC1683.11c	icl1	isocitrate lyase Icl1	1.016646531	1.7176E-13
SPBC83.09c	lin1	U5 snRNP subunit Snu40	1.013317715	5.1521E-06
SPAC1B3.06c		UbiE family methyltransferase	1.01138584	1.8637E-08
SPBC359.05	abc3	vacuolar heme ABC transmembrane exporter Abc3	1.006487101	4.0067E-12
SPCC191.11	inv1	external invertase, beta-fructofuranosidase Inv1	1.004688243	7.7111E-08
