# Supplementary material for: Suppression of inositol pyrophosphate toxicosis and hyper-repression of the fission yeast PHO regulon by loss-of-function mutations in chromatin remodelers Snf22 and Sol1
Source: mBio. 2024 Jun 20;15(7):e01252-24. doi: 10.1128/mbio.01252-24 (PMC11253589; doi:10.1128/mbio.01252-24)
Supplement: Table S4 — Coding genes dysregulated in snf22 ATPase mutant. [file mbio.01252-24-s0003.pdf]

Table S4 legend.

Lists of the protein coding genes that were downregulated by at least 2-fold (log2 change of –1.0 or greater) in *snf22-(D996A-E997A)* cells and the protein-coding genes that were upregulated by at least 2-fold (log2 change of 1.0 or greater) in *snf22-(D996A-E997A)* cells. Genes that were downregulated or upregulated by at least 4-fold (log2 changes of at least –2.0 or 2.0, respectively) are shaded.

Systematic ID	Gene name	Product description	log2FoldChange	pvalue
SPBPB2B2.01		amino acid transmembrane transporter	-8.356126503	8.13087E-97
SPBPB2B2.06c		extracellular 5'-nucleotidase, human NT5E family	-7.788758116	2.08196E-44
SPBP4G3.02	pho1	extracellular acid phosphatase Pho1	-6.772538132	0
SPBC8E4.01c	pho84	plasma membrane inorganic phosphate transmembrane transporter	-6.326600083	0
SPAC17D4.01	pex7	peroxin-7	-5.94098249	0
SPBPB2B2.05		class I glutamine amidotransferase family protein	-4.250142913	1.07808E-28
SPBC23G7.13c		plasma membrane urea transmembrane transporter	-4.190298107	7.04058E-30
SPAC1039.02		extracellular 5'-nucleotidase, human NT5E family	-3.994929537	6.13186E-55
SPAPB24D3.07c		Schizosaccharomyces pombe specific protein	-3.719690511	1.8547E-128
SPBC1289.14		adducin	-3.356776162	1.06271E-24
SPAC2E1P3.05c		fungal cellulose binding domain protein	-3.228658614	1.7239E-153
SPCC794.04c		amino acid transmembrane transporter	-3.118162755	3.20543E-35
SPBC8E4.12c	ecl3	extender of chronological lifespan protein Ecl3	-3.000827606	1.31507E-54
SPBC29B5.02c	isp4	plasma membrane OPT oligopeptide transmembrane transporter family Isp4	-2.943313412	4.6392E-59
SPCC1223.03c	gut2	glycerol-3-phosphate dehydrogenase Gut2	-2.787249519	4.63473E-58
SPAC3C7.14c	obr1	NAD(P)H dehydrogenase (quinone)	-2.755263341	5.6447E-155
SPCC757.07c	ctt1	catalase	-2.562013835	2.39233E-50
SPBC1861.02	abp2	unknown protein, may bind replication origins Abp2	-2.529190937	7.4394E-108
SPCC794.12c	mae2	malic enzyme, malate dehydrogenase (oxaloacetate decarboxylating), Mae2	-2.502075859	2.4303E-143
SPBC1271.07c		N-acetyltransferase	-2.411494344	9.1491E-76
SPAC15E1.02c		DUF1761 family protein	-2.221722834	5.5835E-29
SPBC1685.17		Schizosaccharomyces pombe specific protein	-2.147293715	6.0686E-33
SPBC1271.08c		Schizosaccharomyces pombe specific protein	-2.005463584	1.6022E-36
SPBC1861.01c	cnp3	CENP-C ortholog Cnp3	-1.985182779	1.12738E-38
SPAC5H10.06c	adh4	alcohol dehydrogenase Adh4	-1.937942313	2.1553E-57
SPAC1002.16c		carboxylic acid transmembrane transporter	-1.933520394	2.39923E-32
SPBC16E9.16c	lsd90	Lsd90 protein	-1.860603027	3.15352E-08
SPCC622.12c	gdh1	NADP-specific glutamate dehydrogenase Gdh1	-1.858249815	8.7298E-102
SPBC19C7.04c		DUF2406 family conserved fungal protein	-1.842826057	3.96773E-11
SPCC70.08c		methyltransferase	-1.838678286	4.77918E-10
SPAC1B3.16c	vht1	plasma membrane vitamin H transmembrane transporter Vht1	-1.716457298	2.762E-172
SPACUNK4.17		NAD binding dehydrogenase family protein	-1.671540118	2.39598E-23
SPBP8B7.05c	nce103	carbonic anhydrase	-1.66829778	4.98708E-49
SPAC1039.01		amino acid transmembrane transporter	-1.66679612	1.01133E-50
SPCC584.16c		Schizosaccharomyces specific protein	-1.632516883	1.09816E-50
SPBC24C6.09c		phosphoketolase family protein	-1.587719857	1.93471E-09
SPAC57A7.05		transmembrane transporter	-1.558210435	3.50346E-08
SPAC521.03		short chain dehydrogenase, human DHRS7 family	-1.532314492	3.43514E-27
SPAC9.10	thi9	plasma membrane thiamine/proton high affinity transmembrane transporter Thi9	-1.482758396	4.44894E-31
SPCC1223.13	cbf12	DNA-binding transcription factor, CBF1/Su(H)/LAG-1 family Cbf12	-1.480756602	1.62754E-39
SPBPJ4664.02		crazy cell surface glycoprotein	-1.448702904	5.2755E-26
SPAC1002.17c	urg2	uracil phosphoribosyltransferase	-1.441403856	1.92077E-06
SPBC16A3.13	meu7	alpha-amylase homolog Aah4	-1.424995102	1.65277E-13
SPAC110.01	ppk1	serine/threonine protein kinase Ppk1	-1.421494609	2.29815E-38
SPBC336.08	spc24	NMS complex subunit Spc24	-1.420442235	0.000661205
SPAC513.07		flavonol reductase/cinnamoyl-CoA reductase family	-1.416225921	2.10692E-53
SPBC1711.15c		Schizosaccharomyces pombe specific protein	-1.397153953	4.60766E-14
SPCC70.12c	ecl1	extender of chronological lifespan protein Ecl1	-1.383665006	4.75904E-07
SPCPB1C11.03		cysteine transmembrane transporter	-1.355151657	1.51554E-37
SPAC1786.02		phospholipase	-1.339780975	1.49699E-38
SPAC1002.19	urg1	GTP cyclohydrolase II Urg1	-1.338357971	3.64838E-06
SPAC23H3.13c	gpa2	heterotrimeric G protein alpha-2 subunit Gpa2	-1.331225318	1.93021E-29
SPAC29B12.10c	pgt1	plasma membrane glutathione transmembrane transporter Pgt1	-1.311487559	1.26708E-28
SPBC1773.12		DNA-binding transcription factor, zf-fungal binuclear cluster type	-1.309494418	6.13706E-09
SPBC1271.09	tgp1	plasma membrane glycerophosphodiester transmembrane transporter	-1.296183353	1.00912E-15
SPAC1093.01	ppr5	mitochondrial PPR repeat protein Ppr5	-1.286948589	1.67016E-27
SPCC1795.06	map2	P-factor pheromone Map2	-1.275849281	9.7237E-12
SPAC1687.16c	erg31	C-5 sterol desaturase Erg31	-1.27535402	3.32607E-37
SPAC21E11.04	aca1	L-azetidine-2-carboxylic acid acetyltransferase Aca1	-1.265877251	1.1349E-14
SPAC5H10.03		phosphoglycerate mutase/6-phosphofructo-2-kinase family	-1.258420853	4.78067E-24
SPBC409.08		spermine family transmembrane transporter	-1.240904572	1.76625E-35
SPBC1685.13	fhn1	eisosome assembly protein Fhn1	-1.236261609	0.000146457
SPAC2H10.01		DNA-binding transcription factor, zf-fungal binuclear cluster type	-1.234543755	5.26091E-11
SPBC530.11c		DNA-binding transcription factor, zf-fungal binuclear cluster type	-1.217915618	2.98282E-49
SPAC31G5.11	pac2	cAMP-independent regulatory protein Pac2	-1.214303995	1.79528E-35
SPAC1399.04c	uck2	uracil phosphoribosyltransferase Uck2	-1.208067358	1.42571E-11
SPBC839.08c	its8	pig-N	-1.201432431	3.69628E-23
SPBC1683.01	pho841	plasma membrane inorganic phosphate transmembrane transporter Pho841	-1.198677426	1.2708E-41
SPCC1223.08c	dfr1	dihydrofolate reductase/ lysophospholipase fusion protein Dfr1	-1.182784786	1.26802E-31
SPBC887.17		plasma membrane guanine and adenine transmembrane transporter	-1.162654045	3.4292E-53
SPBC428.05c	arg12	argininosuccinate synthase Arg12	-1.144694042	6.39989E-24
SPBC215.08c	arg4	arginine specific carbamoyl-phosphate synthase Arg4	-1.144102674	1.52922E-22
SPAC7D4.08		Schizosaccharomyces pombe specific protein	-1.141640088	7.65482E-09
SPBC354.12	gpd3	glyceraldehyde 3-phosphate dehydrogenase Gpd3	-1.131616546	3.8969E-13
SPBC1198.02	dea2	adenine deaminase Dea2	-1.087720886	6.92668E-33
SPAC11D3.17		DNA-binding transcription factor, zf-fungal binuclear cluster type	-1.085973472	2.43327E-11
SPBC1703.08c	fau1	5-formyltetrahydrofolate cyclo-ligase Fau1	-1.082352692	7.64287E-05
SPAPB24D3.09c	pdr1	ABC transmembrane transporter Pdr1	-1.070709227	3.13457E-05
SPBC8E4.03		agmatinase 2	-1.065271564	1.3035E-19
SPAC2E1P5.02c	mug109	Rab GTPase binding protein upregulated in meiosis II	-1.056958958	1.00269E-24
SPBC56F2.09c	arg5	arginine specific carbamoyl-phosphate synthase subunit Arg5	-1.054689122	3.27916E-13
SPAC8E11.10		sorbose reductase	-1.053646238	3.72882E-31
SPAC821.09	eng1	cell septum surface endo-1,3-beta-glucanase Eng1	-1.049571833	3.41218E-15
SPAC323.07c		MatE family transmembrane transporter	-1.037086778	2.20936E-24
SPAC22A12.06c	fsh2	serine hydrolase-like, human TSTD2 and OVCA2 ortholog	-1.033690061	5.4063E-60
SPCC965.13		plasma membrane pyridoxal family transmembrane transporter	-1.033271403	2.58116E-46
SPAC1002.18	urg3	DUF1688 family fungal conserved protein, implicated in uracil or riboflavin metabolism	-1.010326124	3.37796E-10

Systematic ID	Gene name	Product description	log2FoldChange	pvalue
SPAC1F8.03c	str3	plasma membrane heme transmembrane transporter Str3	6.453067184	1.85546E-24
SPCC548.07c	ght1	plasma membrane high-affinity glucose:proton symporter Ght1	4.690540162	0
SPBC359.06	mug14	adducin, involved in actin cytoskeleton organization	3.972384015	1.81289E-36
SPCC1235.18		dubious	3.631882829	1.3303E-118
SPBPB21E7.11		Schizosaccharomyces pombe specific protein	3.517671991	1.00602E-22
SPBC359.02	alr2	alanine racemase Alr2	3.432960082	7.03991E-92
SPCC1235.14	ght5	plasma membrane high-affinity glucose/fructose:proton symporter Ght5	3.428104344	1.6768E-134
SPBC1683.08	ght4	plasma membrane hexose:proton symporter, unknown specificity Ght4	3.051460496	6.7285E-223
SPCC1235.17		dubious	3.020996933	5.8683E-105
SPAPB8E5.05	mfm1	M-factor precursor Mfm1	2.837086434	1.25217E-33
SPBPB21E7.04c	cmt2	O-methyltransferase, human COMT catechol homolog 2	2.827812656	8.833E-11
SPBC1348.14c	ght7	plasma membrane hexose transmembrane transporter Ght7	2.709886088	1.23745E-17
SPAC11H11.04	mam2	pheromone p-factor receptor Mam2	2.673000551	6.62392E-52
SPAC1F8.01	ght3	plasma membrane gluconate:proton symporter Ght3	2.240794698	1.46228E-07
SPBC1683.09c	frp1	plasma membrane ferric-chelate reductase Frp1	2.203041581	1.59713E-26
SPAC1F7.08	fio1	plasma membrane iron transport multicopper oxidase Fio1	2.175251617	1.01042E-37
SPBC4F6.09	str1	plasma membrane siderophore-iron transmembrane transporter Str1	2.023464653	5.32595E-12
SPBPB10D8.03		pseudogene transporter	2.017690751	4.36603E-16
SPAC4F8.08	mug114	Schizosaccharomyces pombe specific protein Mug114	1.989119681	3.26962E-13
SPCC1739.08c		short chain dehydrogenase	1.979907813	2.6235E-18
SPAC1A6.04c	plb1	phospholipase B homolog Plb1	1.972146806	3.096E-59
SPCC1840.12	opt3	OPT oligopeptide transmembrane transporter family protein Opt3	1.951925029	5.9855E-16
SPAC56F8.14c	mug115	Schizosaccharomyces pombe specific protein Mug115	1.816525398	2.28718E-58
SPBC56F2.06	mug147	Schizosaccharomyces specific protein Mug147	1.786222351	9.26609E-13
SPBC660.16	gnd1	phosphogluconate dehydrogenase, decarboxylating	1.77533545	3.83583E-71
SPAC11E3.06	map1	DNA-binding transcription factor, MADS-box Map1	1.763535619	4.04636E-16
SPBPB10D8.02c		arylsulfatase	1.760588625	7.09142E-18
SPAC1F7.07c	fip1	plasma membrane iron transmembrane transporter Fip1	1.738970725	3.1157E-11
SPBC359.05	abc3	vacuolar heme ABC transmembrane exporter Abc3	1.703697209	6.02988E-32
SPBC19C2.06c	mug124	Schizosaccharomyces pombe specific protein	1.678991332	7.79636E-42
SPBPB2B2.10c	gal7	galactose-1-phosphate uridylyltransferase Gal7	1.656124461	1.19626E-29
SPBPB21E7.01c	eno102	enolase	1.651597267	1.2937E-10
SPBC215.10	odr1	HAD superfamily hydrolase, unknown role	1.645438275	2.79247E-78
SPCC1906.04	wtf20	wtf antidote-like meiotic drive suppressor Wtf20	1.613888037	2.18209E-10
SPAC22F8.04	pet1	Golgi phosphoenolpyruvate transmembrane transporter Pet1	1.558133835	6.91043E-61
SPBPB2B2.12c	gal10	UDP-glucose 4-epimerase/aldose 1-epimerase Gal10	1.50924712	9.18924E-19
SPAC56F8.13		dubious	1.486093409	1.80427E-12
SPAC20G4.03c	hri1	eIF2 alpha kinase Hri1	1.47307458	4.04297E-40
SPAC17A2.11		dubious	1.471635353	1.1454E-11
SPBC23G7.10c		NADH-dependent flavin oxidoreductase	1.462046818	2.84778E-19
SPAC9E9.09c	atd1	aldehyde dehydrogenase	1.378846263	4.9604E-16
SPBP4H10.10	rbd3	mitochondrial rhomboid family protease	1.363796708	6.33738E-18
SPAC4H3.03c		glucan 1,4-alpha-glucosidase	1.363476412	1.16691E-10
SPCC794.02	wtf5	wtf antidote-like meiotic drive suppressor Wtf5	1.329608111	3.42477E-22
SPAC1A6.06c	meu31	Schizosaccharomyces specific protein Meu31	1.321256422	1.90325E-11
SPCC4F11.05		dubious	1.318079957	6.57077E-09
SPAC110.05	SPAC110.06	dubious	1.315603942	3.64752E-19
SPBC1A4.06c	tam41	mitochondrial phosphatidate cytidylyltransferase Tam41	1.29427054	8.34848E-17
SPBC1348.12		DNA-binding transcription factor	1.244907137	1.15806E-06
SPAC1565.04c	ste4	MAPK cascade adaptor protein Ste4	1.226814054	8.70873E-07
SPCC330.04c	mug135	DUF1773 family protein, with repeat expansion	1.200120791	5.33977E-06
SPCC794.03		amino acid transmembrane transporter	1.176513446	4.76177E-15
SPBPB2B2.13	gal1	galactokinase Gal1	1.171223039	5.82966E-19
SPAC3G9.11c	pdc201	pyruvate decarboxylase	1.167485598	5.39052E-15
SPAC23D3.12	pho842	plasma membrane inorganic phosphate transmembrane transporter Pho842	1.136553554	1.21261E-17
SPBC32H8.02c	nep2	NEDD8 protease Nep2	1.096014627	1.26618E-23
SPCC4F11.04c	imt2	mannosyltransferase Imt2	1.091946847	4.45038E-13
SPAC26H5.08c	bgl2	glucan beta-glucosidase Bgl2	1.08810474	2.17583E-19
SPBC359.03c	aat1	plasma membrane amino acid transmembrane transporter Aat1	1.083941121	2.20165E-21
SPAC6C3.08	nas6	proteasome assembly chaperone, gankyrin	1.083245655	3.76528E-14
SPCC1020.03	mmt1	mitochondrial iron ion transmembrane transporter Mmt1	1.078334135	3.25592E-14
SPAC688.06c	slx4	structure-specific endonuclease subunit Slx4	1.072337522	1.22112E-05
SPAC57A10.06	mug15	Schizosaccharomyces specific protein Mug15	1.058510042	4.09128E-07
SPCC320.14	sry1	serine racemase Sry1	1.051806856	8.58343E-25
SPAC11D3.09		agmatinase	1.041975961	2.61955E-10
SPCC790.03	rbd2	Golgi rhomboid protease Rbd2	1.039302518	9.3399E-26
SPBC18A7.01	xpa1	X-Pro dipeptidase	1.031224744	2.10825E-21
SPAC27D7.08c	mtl16	23S rRNA/U6 snRNA (adenine-N(6))-methyltransferase Mtl16	1.026028615	5.25717E-09
SPBC106.02c	srx1	sulfiredoxin	1.022399887	4.12753E-05
SPAC26F1.05	mug106	Schizosaccharomyces pombe specific protein Mug106	1.01777641	3.06381E-20
SPAC3A12.02		mitochondrial inorganic diphosphatase	1.004338934	9.29547E-08
SPBC8D2.05c	sfi1	spindle pole body half bridge protein Sfi1	1.00277775	1.08271E-08
SPBPB21E7.08		pseudogene	1.001311862	1.71128E-22
