# Supplementary material for: Suppression of inositol pyrophosphate toxicosis and hyper-repression of the fission yeast PHO regulon by loss-of-function mutations in chromatin remodelers Snf22 and Sol1
Source: mBio. 2024 Jun 20;15(7):e01252-24. doi: 10.1128/mbio.01252-24 (PMC11253589; doi:10.1128/mbio.01252-24)
Supplement: Table S5 — Overlap of dysregulated coding genes between snf22∆ and snf22 ATPase mutants. [file mbio.01252-24-s0004.pdf]

Table S5 legend.

Lists of the protein coding genes that were downregulated by at least 2-fold (log2 change of –1.0 or greater) in *snf22*Δ cells and *snf22-(D996A-E997A)* cells and the protein-coding genes that were upregulated by at least 2-fold (log2 change of 1.0 or greater) in *snf22*Δ cells and *snf22-(D996A-E997A)* cells.

Systematic ID	Gene name	Product description	snf22Δ	snf22-D996A-E997A
SPBPB2B2.01		amino acid transmembrane transporter	-7.19	-8.36
SPBPB2B2.06c		extracellular 5'-nucleotidase, human NT5E family	-7.07	-7.79
SPAC17D4.01	pex7	peroxin-7	-5.94	-5.94
SPBP4G3.02	pho1	extracellular acid phosphatase Pho1	-5.80	-6.77
SPAC1039.02		extracellular 5'-nucleotidase, human NT5E family	-4.76	-3.99
SPBC8E4.01c	pho84	plasma membrane inorganic phosphate transmembrane transporter	-4.14	-6.33
SPAC2E1P3.05c		fungal cellulose binding domain protein	-3.60	-3.23
SPBPB2B2.05		class I glutamine amidotransferase family protein	-3.27	-4.25
SPBC29B5.02c	isp4	plasma membrane OPT oligopeptide transmembrane transporter family Isp4	-3.09	-2.94
SPAPB24D3.07c		Schizosaccharomyces pombe specific protein	-2.87	-3.72
SPAC3C7.14c	obr1	NAD(P)H dehydrogenase (quinone)	-2.70	-2.76
SPBC23G7.13c		plasma membrane urea transmembrane transporter	-2.61	-4.19
SPBC1861.02	abp2	unknown protein, may bind replication origins Abp2	-2.45	-2.53
SPAC513.07		flavonol reductase/cinnamoyl-CoA reductase family	-2.43	-1.42
SPBC1271.08c		Schizosaccharomyces pombe specific protein	-2.40	-2.01
SPCPB1C11.03		cysteine transmembrane transporter	-2.31	-1.36
SPCC70.08c		methyltransferase	-2.30	-1.84
SPAC110.01	ppk1	serine/threonine protein kinase Ppk1	-2.18	-1.42
SPAC5H10.03		phosphoglycerate mutase/6-phosphofructo-2-kinase family	-2.10	-1.26
SPCC584.16c		Schizosaccharomyces specific protein	-2.05	-1.63
SPCC1223.03c	gut2	glycerol-3-phosphate dehydrogenase Gut2	-2.02	-2.79
SPAC1002.16c		carboxylic acid transmembrane transporter	-1.97	-1.93
SPBC8E4.12c	ecl3	extender of chronological lifespan protein Ecl3	-1.86	-3.00
SPBC1861.01c	cnp3	CENP-C ortholog Cnp3	-1.82	-1.99
SPAC29B12.10c	pgt1	plasma membrane glutathione transmembrane transporter Pgt1	-1.75	-1.31
SPBC1289.14		adducin	-1.74	-3.36
SPAC5H10.06c	adh4	alcohol dehydrogenase Adh4	-1.69	-1.94
SPCC757.07c	ctt1	catalase	-1.68	-2.56
SPAC8E11.10		sorbose reductase	-1.64	-1.05
SPAC7D4.08		Schizosaccharomyces pombe specific protein	-1.60	-1.14
SPBP8B7.05c	nce103	carbonic anhydrase	-1.59	-1.67
SPBC1271.07c		N-acetyltransferase	-1.58	-2.41
SPAC1039.01		amino acid transmembrane transporter	-1.56	-1.67
SPBC1685.17		Schizosaccharomyces pombe specific protein	-1.52	-2.15
SPBC1685.13	fhn1	eisosome assembly protein Fhn1	-1.50	-1.24
SPAC21E11.04	aca1	L-azetidine-2-carboxylic acid acetyltransferase Aca1	-1.49	-1.27
SPCC794.12c	mae2	malic enzyme, malate dehydrogenase (oxaloacetate decarboxylating), Mae2	-1.49	-2.50
SPBC24C6.09c		phosphoketolase family protein	-1.46	-1.59
SPBPJ4664.02		crazy cell surface glycoprotein	-1.43	-1.45
SPCC70.12c	ecl1	extender of chronological lifespan protein Ecl1	-1.41	-1.38
SPAC1002.17c	urg2	uracil phosphoribosyltransferase	-1.39	-1.44
SPAC1687.16c	erg31	C-5 sterol desaturase Erg31	-1.39	-1.28
SPAC31G5.11	pac2	cAMP-independent regulatory protein Pac2	-1.37	-1.21
SPAC821.09	eng1	cell septum surface endo-1,3-beta-glucanase Eng1	-1.36	-1.05
SPAC9.10	thi9	plasma membrane thiamine/proton high affinity transmembrane transporter	-1.34	-1.48
SPBC336.08	spc24	NMS complex subunit Spc24	-1.34	-1.42
SPAC23H3.13c	gpa2	heterotrimeric G protein alpha-2 subunit Gpa2	-1.34	-1.33
SPAC1B3.16c	vht1	plasma membrane vitamin H transmembrane transporter Vht1	-1.32	-1.72
SPAPB24D3.09c	pdr1	ABC transmembrane transporter Pdr1	-1.29	-1.07
SPBC428.05c	arg12	argininosuccinate synthase Arg12	-1.27	-1.14
SPBC1711.15c		Schizosaccharomyces pombe specific protein	-1.27	-1.40
SPAC11D3.17		DNA-binding transcription factor, zf-fungal binuclear cluster type	-1.24	-1.09
SPAC1093.01	ppr5	mitochondrial PPR repeat protein Ppr5	-1.21	-1.29
SPBC1198.02	dea2	adenine deaminase Dea2	-1.20	-1.09
SPCC1223.13	cbf12	DNA-binding transcription factor, CBF1/Su(H)/LAG-1 family Cbf12	-1.19	-1.48
SPAC57A7.05		transmembrane transporter	-1.17	-1.56
SPCC965.13		plasma membrane pyridoxal family transmembrane transporter	-1.13	-1.03
SPBC1683.01	pho841	plasma membrane inorganic phosphate transmembrane transporter Pho841	-1.12	-1.20
SPAC1002.18	urg3	DUF1688 family fungal protein, implicated in uracil or riboflavin metabolism	-1.11	-1.01
SPBC215.08c	arg4	arginine specific carbamoyl-phosphate synthase Arg4	-1.10	-1.14
SPBC56F2.09c	arg5	arginine specific carbamoyl-phosphate synthase subunit Arg5	-1.10	-1.05
SPBC16E9.16c	lsd90	Lsd90 protein	-1.10	-1.86
SPBC19C7.04c		DUF2406 family conserved fungal protein	-1.09	-1.84
SPAC22A12.06c	fsh2	serine hydrolase-like, human TSTD2 and OVCA2 ortholog	-1.08	-1.03
SPAC521.03		short chain dehydrogenase, human DHRS7 family	-1.07	-1.53
SPBC887.17		plasma membrane guanine and adenine transmembrane transporter	-1.01	-1.16
SPAC1399.04c	uck2	uracil phosphoribosyltransferase Uck2	-1.00	-1.21

Systematic ID	Gene name	Product description	snf22Δ	snf22-D996A-E997A
SPBC359.06	mug14	adducin, involved in actin cytoskeleton organization	5.845140283	3.972384015
SPCC1739.08c		short chain dehydrogenase	5.688935216	1.979907813
SPBC1683.08	ght4	plasma membrane hexose:proton symporter, unknown specificity	5.217563544	3.051460496
SPCC548.07c	ght1	plasma membrane high-affinity glucose:proton symporter Ght1	5.098431656	4.690540162
SPBC359.02	alr2	alanine racemase Alr2	4.919821835	3.432960082
SPAC1F8.01	ght3	plasma membrane gluconate:proton symporter Ght3	4.758052367	2.240794698
SPCC1235.17		dubious	4.399533214	3.020996933
SPCC1235.18		dubious	4.112958153	3.631882829
SPCC1235.14	ght5	plasma membrane high-affinity glucose/fructose:proton symporter Ght5	3.996080451	3.428104344
SPBPB2B2.12c	gal10	UDP-glucose 4-epimerase/aldose 1-epimerase Gal10	2.953863355	1.50924712
SPBP4H10.10	rbd3	mitochondrial rhomboid family protease	2.495921243	1.363796708
SPAC1A6.04c	plb1	phospholipase B homolog Plb1	2.44163559	1.972146806
SPBC56F2.06	mug147	Schizosaccharomyces specific protein Mug147	2.189273124	1.786222351
SPAC4H3.03c		glucan 1,4-alpha-glucosidase	2.174071608	1.363476412
SPAC17A2.11		dubious	2.149264058	1.471635353
SPBPB2B2.10c	gal7	galactose-1-phosphate uridylyltransferase Gal7	2.054184062	1.656124461
SPBPB2B2.13	gal1	galactokinase Gal1	1.97773818	1.171223039
SPBC32H8.02c	nep2	NEDD8 protease Nep2	1.922660976	1.096014627
SPCC1840.12	opt3	OPT oligopeptide transmembrane transporter family protein Opt3	1.863590083	1.951925029
SPAC1F7.08	fio1	plasma membrane iron transport multicopper oxidase Fio1	1.823577771	2.175251617
SPCC4F11.05		dubious	1.728322498	1.318079957
SPBC215.10	odr1	HAD superfamily hydrolase, unknown role	1.671134265	1.645438275
SPCC794.02	wtf5	wtf antidote-like meiotic drive suppressor Wtf5	1.592667017	1.329608111
SPAC1A6.06c	meu31	Schizosaccharomyces specific protein Meu31	1.410605518	1.321256422
SPCC330.04c	mug135	DUF1773 family protein, with repeat expansion	1.408782069	1.200120791
SPBC660.16	gnd1	phosphogluconate dehydrogenase, decarboxylating	1.406291301	1.77533545
SPCC1906.04	wtf20	wtf antidote-like meiotic drive suppressor Wtf20	1.391602597	1.613888037
SPBPB21E7.04c	cmt2	O-methyltransferase, human COMT catechol homolog 2	1.37351221	2.827812656
SPAC20G4.03c	hri1	eIF2 alpha kinase Hri1	1.361998082	1.47307458
SPBC23G7.10c		NADH-dependent flavin oxidoreductase	1.334726787	1.462046818
SPAC56F8.14c	mug115	Schizosaccharomyces pombe specific protein Mug115	1.311844458	1.816525398
SPCC794.03		amino acid transmembrane transporter	1.304561729	1.176513446
SPBC19C2.06c	mug124	Schizosaccharomyces pombe specific protein	1.280945195	1.678991332
SPAC11D3.09		agmatinase	1.280397855	1.041975961
SPAC3G9.11c	pdc201	pyruvate decarboxylase	1.119990762	1.167485598
SPCC320.14	sry1	serine racemase Sry1	1.101744138	1.051806856
SPAC3A12.02		mitochondrial inorganic diphosphatase	1.098679608	1.004338934
SPAC6C3.08	nas6	proteasome assembly chaperone, gankyrin	1.06350406	1.083245655
SPBC1348.12		DNA-binding transcription factor	1.0568035	1.244907137
SPAC27D7.08c	mtl16	23S rRNA/U6 snRNA (adenine-N(6))-methyltransferase Mtl16	1.050295941	1.026028615
SPBC1A4.06c	tam41	mitochondrial phosphatidate cytidylyltransferase Tam41	1.034104164	1.29427054
SPBC359.05	abc3	vacuolar heme ABC transmembrane exporter Abc3	1.006487101	1.703697209
